# The HamE scaffold positively regulates MpkB phosphorylation to promote development and secondary metabolism in *Aspergillus nidulans*

**DOI:** 10.1038/s41598-018-34895-6

**Published:** 2018-11-08

**Authors:** Dean Frawley, Betim Karahoda, Özlem Sarikaya Bayram, Özgür Bayram

**Affiliations:** 1 Biology Department, Maynooth University, Maynooth, Co. Kildare, Ireland; 2 Maynooth University Human Health Research Institute, Kildare, Ireland

## Abstract

Mitogen-activated protein kinase (MAPK) pathways are conserved signalling cascades in eukaryotes which regulate a myriad of processes in fungi from sexual reproduction to stress responses. These pathways rely on recruitment of three kinases on a scaffold protein to facilitate efficient kinase phosphorylation and subsequent downstream signalling to the nucleus. The model filamentous fungus *Aspergillus nidulans* utilises a MAPK pathway termed the pheromone module to regulate both development and secondary metabolism. This complex consists of the MAP3K (SteC), MAP2K (MkkB), MAPK (MpkB) and adaptor protein SteD. To date, there has been no scaffold protein identified for this MAPK pathway. In this study, we characterised a protein termed HamE, which we propose as a scaffold that regulates kinase phosphorylation and signalling in the pheromone module. Mass spectrometry analysis and BIFC experiments revealed that HamE physically interacts with both MkkB and MpkB and transiently interacts with SteC. Deletion of *hamE* or any of the pheromone module kinases results in reduced sporulation and complete abolishment of cleistothecia production. Mutants also exhibited reductions in expression of secondary metabolite gene clusters, including the velvet complex and sterigmatocystin genes. HamE acts as a positive regulator of MpkB phosphorylation, allowing for HamE to subsequently regulate development and secondary metabolism.

## Introduction

For eukaryotic organisms to rapidly respond to the myriad of environmental stimuli they encounter, an array of protein signalling cascades are utilised^1,2^. Examples of conserved signalling pathways in eukaryotes are mitogen-activated protein kinase (MAPK) cascades^3^. These signalling cascades consist of three protein kinases (MAPKKK, MAPKK and MAPK) that phosphorylate one another, downstream of a receptor. The terminal MAPK becomes dually phosphorylated at a conserved Thr-X-Tyr motif and translocates into the nucleus where it activates specific transcription factors^4–6^. MAPK cascades regulate a range of processes in eukaryotes from fungi to humans. In mammals, there are 3 main MAPK families, the ERKs, JNKs and p38/SAPKs. These kinases are involved in cell growth, response to environmental stresses and immune system regulation respectively^7–10^. In yeast, 5 MAPKs (Fus3, Kss1, Hog1, Slt2/Mpk1 and Smk1) have been identified. These kinases regulate the pheromone response, filamentous growth, osmotic response, polarized cell growth and spore wall assembly pathways respectively^11–15^. The most extensively studied of the yeast MAP kinase pathways is the Fus3 module, which stimulates cell mating in response to pheromone detection^11^. Upon binding of pheromones to G-protein coupled receptors (GPCRs) at the plasma membrane, the Gβγ subunits of the receptor dissociate and recruit a large multi-domain scaffold protein known as Ste5^16,17^. Ste5 then assembles a three-tiered kinase module consisting of the kinases Ste11 (MAPKKK), Ste7 (MAPKK) and Fus3 (MAPK)^18,19^. Anchoring of Ste11 to the membrane by the adaptor protein Ste50^20,21^ and phosphorylation of Ste11 via the p21 activated kinase Ste20 triggers sequential phosphorylation of each kinase, further amplifying the signal^6^. Upon phosphorylation, Fus3 migrates to the nucleus where it activates the Ste12 transcription factor^22^. This, in turn, regulates sexual development, allowing for two neighbouring yeast cells to fuse^11,23^.

Knowledge of the yeast Fus3 module has led to the discovery of homologous MAP kinases in filamentous fungi, which play roles in processes like conidiation, pathogenesis and secondary metabolite (SM) production^24–26^. The model ascomycete fungus *Aspergillus nidulans* has been extensively studied to gain insight on fungal genetics and development^27–30^. This filamentous fungus is capable of reproducing asexually via the production of haploid spores known as conidia^29^ and sexually via the formation of sexual ascospores enclosed within fruiting bodies known as cleistothecia^31^. Sexual development in *A*. *nidulans* is coupled to SM production via MAPK signalling and a heterotrimeric complex (VeA-VelB-LaeA) known as the velvet complex in the nucleus^32^.

Homologs of the core Fus3 module components have been identified in *A*. *nidulans* like SteC (Ste11), MkkB (Ste7), MpkB (Fus3), SteD (Ste50) and AnSte12/SteA (Ste12). These proteins have been shown to play roles in the regulation of asexual and sexual development as well as SM production^33–37^. It was shown that SteC, MkkB and MpkB associate with the adaptor protein SteD at the plasma membrane to form the *A*. *nidulans* pheromone module. This allows for phosphorylation of MpkB and subsequent translocation of this kinase into the nucleus. MpkB then phosphorylates the SteA transcription factor and the velvet protein VeA to regulate both development and SM production^32,36,38,39^. Despite resemblance of this pathway to the yeast Fus3 module, a Ste5 homolog has not been identified and it appears that Ste5 homologs are absent in filamentous fungi^40^. This proposes the question of how these kinases are assembled in the correct orientation at the membrane, allowing for MpkB phosphorylation and signal propagation to the nucleus.

Another model ascomycete fungus *Neurospora crassa* is commonly used to study fungal genetics and cell development^41,42^. This filamentous fungus utilises a kinase cascade (NRC-1-MEK-2-MAK-2), homologous to the yeast Fus3 module, to regulate germling and hyphal fusion^24,43^. This is essential in filamentous fungi for generating an interconnected network of cells, known as the mycelium^44^. It has been shown that the three-tiered kinase cascade associates with the adaptor protein STE-50 and that STE-50 influences NRC-1 activation^45^, similar to that observed in *A*. *nidulans*. Another similarity to *A*. *nidulans* is that a homolog of yeast Ste5 does not exist in *N*. *crassa*. However, a protein considered essential for cell fusion, known as HAM-5^46,47^, was characterised as a scaffold for the MAK-2 cascade, having been shown to physically associate with all three kinases and the adaptor STE-50^45,48^. This complex localises in puncta at opposing hyphal tips during chemotropic interactions and undergoes cycles of assembly and disassembly^48^. MAK-2 phosphorylation results in activation of PP-1 in the nucleus^45^, a transcription factor similar to yeast Ste12 that regulates cell fusion, sexual development and SM production^24,49^.

It has been shown that HAM-5 is highly conserved in filamentous ascomycete fungi^50^, proposing the question of whether homologs of this protein may act as scaffolds in other species. In this study, we identified the *A*. *nidulans* HAM-5 homolog (HamE) and have shown, via a proteomics approach, that this protein associates with kinases of the *A*. *nidulans* pheromone module. We have also shown that HamE is required for asexual sporulation, sexual cleistothecia formation and production of various SMs. Based on our data, we propose that HamE modulates the phosphorylation states of both MkkB and MpkB, allowing for efficient signalling to the nucleus and regulation of both development and secondary metabolism.

## Results

### The *A*. *nidulans* Ham5 homolog (HamE) interacts with members of the pheromone module

**Figure 1:**
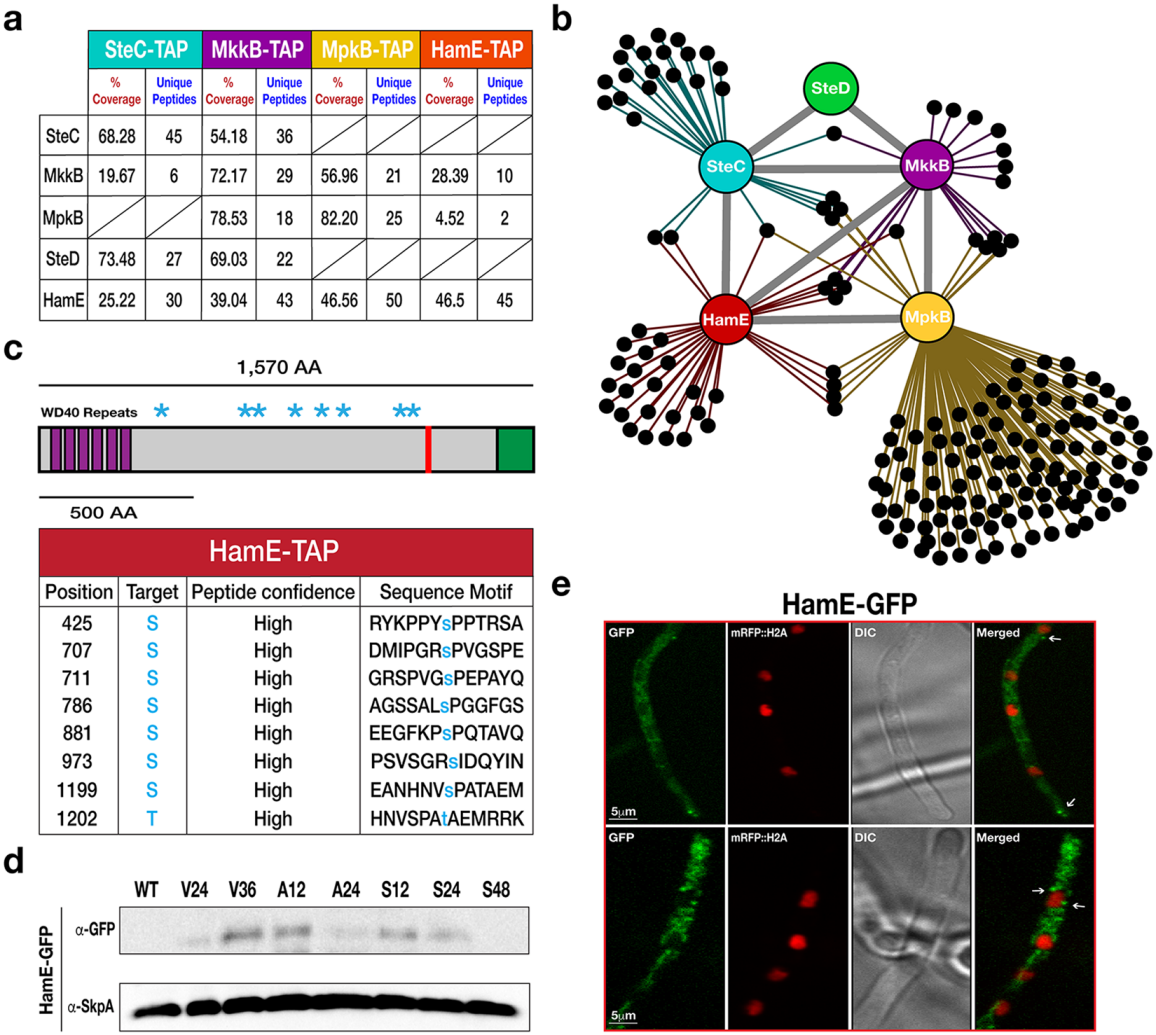
Discovery of the HamE scaffold protein interacting with components of the pheromone module in *A*. *nidulans*. (**a**) TAP pulldowns of the pheromone module kinases and HamE. TAP-tagged proteins are given at the top of the table and co-purified proteins are given on the left-hand side. The percentage of coverage and unique peptides of each detected protein are displayed. 2 biological replicates of each strain were used. (**b**) Interaction network of the pheromone module components based on unique peptides detected in each TAP pulldown. Each black dot represents a protein detected in two independent biological replicates but not in the wild type. (**c**) Schematic overview of the protein structure of HamE. HamE is a large, multi-domain protein that consists of 6 WD40 repeats at it’s N-terminus (aa residues 18–329). The red bar represents a coiled-coil domain (aa residues 1205–1225) and the green shaded area (1479–1570) represents a region of intrinsic protein disorder. Blue stars represent phosphorylation sites detected by mass spectrometry of TAP-tagged HamE. The amino acid positions and residues targeted for phosphorylation are listed in the accompanying table. S (serine), T (Threonine). (**d**) Time course immunoblotting of HamE at various stages of development. V (vegetative), A (asexual), S (sexual). For asexual and sexual induction, the HamE-GFP strain was cultured vegetatively for 24 hours in liquid GMM media and transferred to GMM plates to be incubated in the light and dark respectively. SkpA is used as a loading control. Full-length blots in (**d**) are presented in Supplementary Fig. 2. (**e**) Localisation of HamE-GFP *in vivo* at 16 hours of vegetative growth. The GFP fusion protein is dispersed throughout the cytoplasm and localises at the hyphal tips, cell membrane and nuclear envelope, indicated by white arrows.

In an attempt to identify potential scaffold candidates in the pheromone module, Tandem Affinity Purification (TAP)-tagged SteC, MkkB and MpkB, expressed under their native promoters, were purified and analysed via mass spectrometry (MS). In purifications of each kinase, we detected the uncharacterised *A*. *nidulans* HAM-5 homolog HamE (AN2701) (Fig. 1a,b, Supplementary Tables S1–S3). Reciprocal BLAST searches confirmed that HamE exhibits 62% similarity to HAM-5, with most of this conserved identity existing at the N-terminus. HamE is a large protein (Fig. 1c) of 1,570 amino acids (aa) with 6 putative WD40 repeats at the N-terminus (aa 18–329). A coiled-coil domain was predicted to be located at aa 1205–1225 and a region of intrinsic protein disorder was identified at the C-terminus (aa 1479–1570). HamE was fused to a TAP epitope tag and used for TAP pulldowns and MS analysis to identify potential phosphorylation sites on this protein. A total of 8 putative phosphorylation sites were detected (Fig. 1c, Supplementary Table S5), residing between aa 425–1202. These results complement those found by Jonkers *et al*.^48^ who found that HAM-5 contained similar domains at similar positions to HamE and contained 16 putative phosphorylation sites, suggesting complex methods of regulation exist for Ham proteins.

To determine HamE expression during different developmental stages (Fig. 1d), HamE was fused to a synthetic Green Fluorescent Protein (sGFP) epitope tag and expressed under its native promoter in the *hamE* locus. A time course immunoblotting was performed. Crude protein extracts were isolated from fungal mycelia that were either grown vegetatively in Glucose Minimal Media (GMM) for 24 and 36 hours, asexually (12 and 24 hours) or sexually (12, 24 and 48 hours). It was found that HamE displays complex expression dynamics and is upregulated at the late stages of vegetative growth (36 hours) and early stages of asexual and sexual development (12 hours). HamE appears to be degraded at the late stages of asexual and sexual reproduction, suggesting that it may be required for regulation of the early phases of development.

The HamE-GFP strain was also used to visualise the sub-cellular localisations of HamE via confocal microscopy. In this strain, Histone 2 A (H2A) was tagged with monomeric Red Fluorescent Protein (mRFP) to allow for visualisation of nuclei. It was observed that the HamE-GFP protein is mainly dispersed throughout the cytoplasm but infrequently becomes enriched at the plasma membrane, hyphal tip and nuclear periphery after 16 hours of vegetative growth (Fig. 1e). These patterns of localisation are similar to those observed previously for the pheromone module proteins^36^, suggesting that HamE may interact with the complex at these sites.

To elucidate the interaction network of the pheromone module, TAP-tagged SteC, MkkB, MpkB and HamE expressed under their native promoters were used for TAP pulldowns and MS (Fig. 1a,b). TAP-tagged proteins were isolated from vegetative cultures grown for 24 hours. It was found that TAP of SteC recruited MkkB, HamE and the adaptor protein SteD (Supplementary Table S1), but not MpkB. TAP of MkkB recruited SteC, SteD, MpkB and HamE (Supplementary Table S2). TAP of MpkB recruited MkkB and HamE (Supplementary Table S3) and TAP of HamE recruited MkkB and MpkB (Supplementary Table S4). These interactome data propose a pentameric complex and suggest that HamE physically interacts with the kinases MkkB and MpkB but may only transiently interact with SteC and SteD.

### HamE and the pheromone module proteins contribute to the regulation of asexual and sexual development

**Figure 2:**
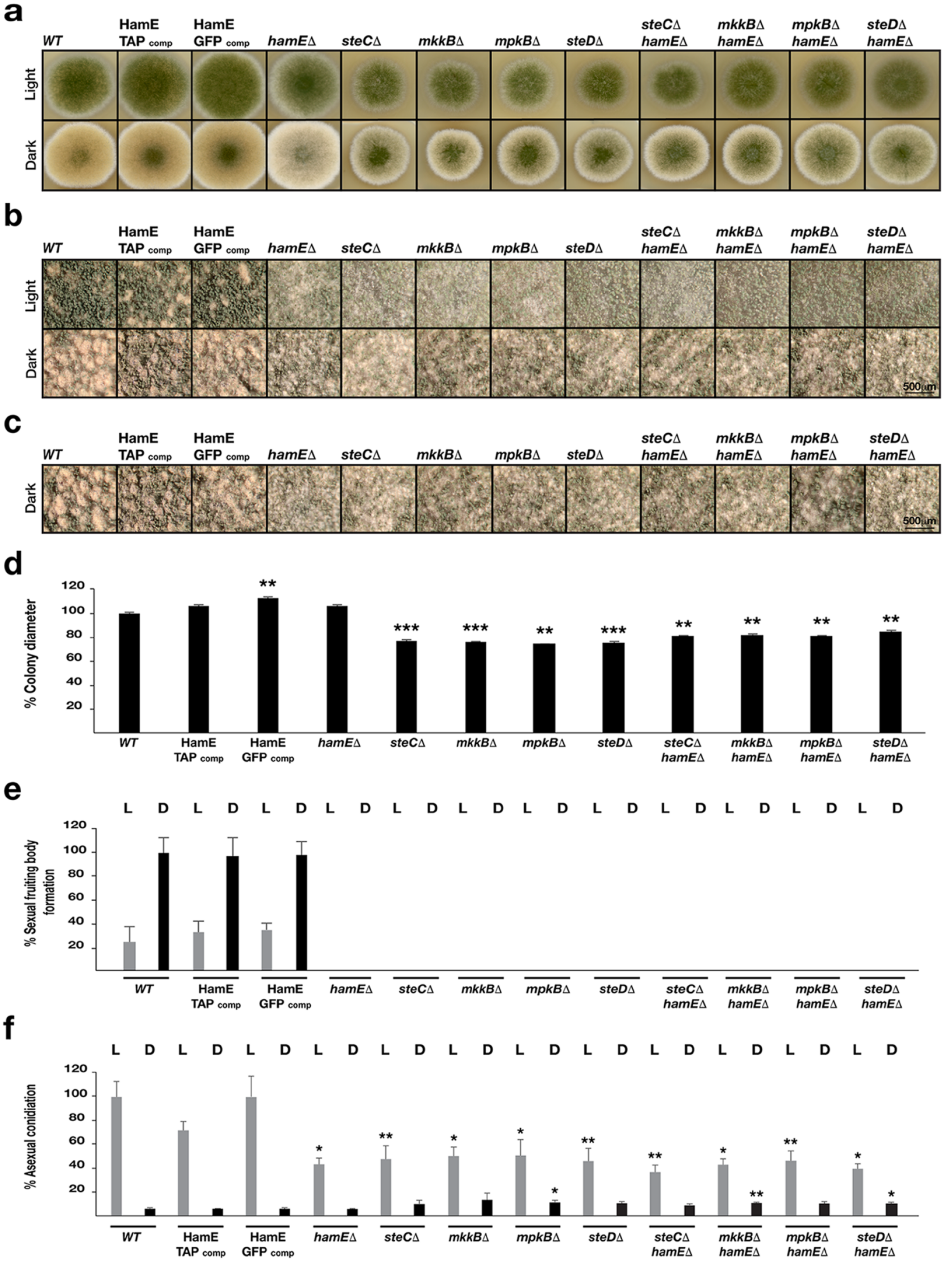
The pheromone module components and HamE are required for asexual conidiation and sexual cleistothecia production in *A*. *nidulans*. (**a**) Vegetative, asexual and sexual phenotypes of deletion strains. Strains were spot inoculated (5 × 10^3^ spores) in triplicate on GMM plates containing supplements and induced asexually (4 days in the light at 37 °C) and sexually (5 days in the dark at 37 °C). (**b**) Close-up stereomicroscopic images of the strains from (**a**). Images were taken at 5x magnification. (**c**) Stereomicroscopic images of sexually induced strains taken at 8x magnification. (**d**) Graphical representation of the colony diameters of each asexually induced strain from (**a**) with respect to the AGB551 wild type strain. Measurements were taken from three independent biological replicates for each strain and the averages were plotted ± s.d. *P-*values were calculated by performing unpaired Student’s *t*-tests (**P < 0.01; ***P < 0.001). (**e**) Quantification of cleistothecia production in each strain. L = light, D = dark. Three 2x magnification images of each asexual and sexual replicate from (**a**) were taken and the cleistothecia were counted (*N* = 9). The averages were plotted ± s.d. as a percentage of the sexually-induced wild type average. (**f**) Quantification of asexual conidiation in each asexually and sexually-induced replicate (*N* = 3) from (**a**). Mean values were plotted ± s.d. as a percentage of the asexually-induced wild type average. *P-*values were calculated as described above (*P < 0.05; **P < 0.01), with light and dark-induced colonies being compared to the respective light and dark-induced wild type colonies.

To determine the influence of the pheromone module and HamE in fungal development, single and double deletion strains were constructed and asexual and sexual development was monitored (Fig. 2). The *hamE* gene was deleted in each pheromone module mutant to generate double deletion strains (Supplementary Fig S1). *hamE*Δ*, steC*Δ*, mkkB*Δ*, mpkB*Δ and *steD*Δ double mutants were created either by replacing the gene open reading frames with the pyridoxine gene (*pyroA*) or the pyrithiamine resistance gene (*ptrA*). Mutants were spot inoculated on GMM agar plates and incubated in the presence and absence of light (continuous illumination under white fluorescent light) for 4 and 5 days respectively (Fig. 2a). *hamE::gfp* and *hamE::tap* fusions, introduced into a *hamE* deletion strain, were also spot-inoculated to show that these fusions are functional.

The colony diameters of all asexually-induced strains (Fig. 2a, upper panel) were measured and the averages of three independent replicates for each strain were plotted as a percentage of the respective wild type average. It was observed that *steC*, *mkkB*, *mpkB* and *steD* mutants all exhibited 20–30% reductions in colony size (Fig. 2d). However, the *hamE* mutant did not show any defects in colony growth and was comparable to the wild type. The quantities of sexual fruiting bodies known as cleistothecia produced by each mutant were determined in comparison to the wild type strain. It was found that all single and double mutants exhibited a pale phenotype (Fig. 2a, lower panel) and showed complete abolishment of sexual development, with no fruiting bodies being produced and only premature aggregates of Hulle cells known as nests being formed (Fig. 2b,c,e). Lastly, asexual conidiation was quantified for each mutant strain, in comparison to the wild type strain. All single and double mutants exhibited a 50–60% reduction in asexual conidiation (Fig. 2a,b upper panels and 2f). The phenotypes of the *hamE* deletion were fully restored by its GFP and TAP fusions (Fig. 2a).

These results show that SteC, MkkB, MpkB and SteD are all required for regulation of colony growth rate, asexual conidiation and sexual cleistothecia development. These data also suggest that HamE may function in a similar manner to the pheromone module components to regulate both asexual and sexual development.

### Expression of various sexual development and SM genes is dependent on the pheromone module proteins and HamE

Given that the pheromone module mutants and the HamE mutant exhibited defects in sexual development, we decided to show the influence of these proteins on the regulation of various sexual development and SM genes. In *A*. *nidulans*, sexual reproduction is co-ordinated with SM production by the heterotrimeric velvet complex (VeA-VelB-LaeA)^32^. *A*. *nidulans* is capable of producing over 40 SMs, that can exhibit beneficial as well as deleterious effects. Examples of SMs produced by *A*. *nidulans* include the carcinogenic Sterigmatocystin (ST), antibiotic Penicillin (PN) and the anti-tumour agent Terrequinone A (TQ)^51^.

**Figure 3:**
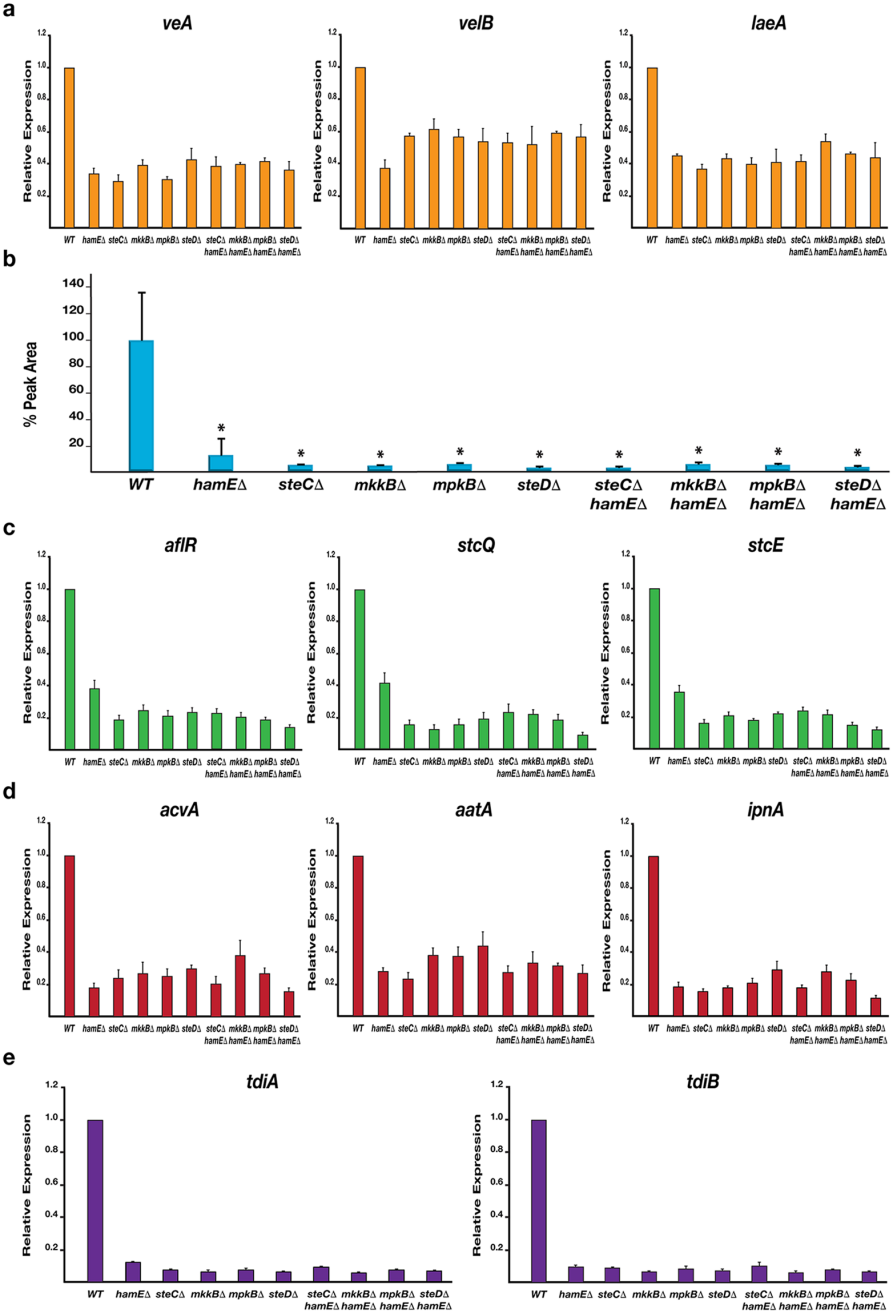
The regulation of various sexual development and secondary metabolism genes is dependent on the pheromone module components and HamE. (**a**) Expression levels of the *veA*, *velB* and *laeA* genes belonging to the velvet complex. Strains were inoculated (5 × 10^6^ spores/ml) in 40 ml of GMM media and incubated for 48 hours at 37 °C on a shaker. For each qPCR experiment (**a**,**c–e**), 2 independent biological replicates and 3 technical replicates were used (*N* = 6) for each strain. The average expression level values were plotted ± s.d. as a percentage of the wild type average. (**b**) HPLC detection of secondary metabolite sterigmatocystin (ST) levels in deletion strains. Strains were inoculated in triplicate and cultured according to the parameters described in (**a**). Average peak area values were plotted as a percentage of the wild type ± s.d. *P-*values were calculated by performing unpaired Student’s *t*-tests (*P < 0.05). (**c**) Expression levels of the *aflR*, *stcQ* and *stcE* genes belonging to the ST gene cluster. (**d**) Expression levels of the *acvA*, *aatA* and *ipnA* genes belonging to the penicillin gene cluster. (**e**) Expression levels of the *tdiA* and *tdiB* genes belonging to the terrequinone A gene cluster.

Real-time/quantitative Polymerase Chain Reaction (qPCR) analysis was performed to determine the relative expression levels of the velvet complex genes *veA*, *velB* and *laeA* in each mutant strain. Reductions in expression of each gene was evident in each mutant (40–70% reduction) in comparison to the wild type (Fig. 3a).

By utilising Reversed-Phase High Performance Liquid Chromatography (RP-HPLC), the levels of ST in each mutant were measured and compared to the wild type (Fig. 3b). It was found that each mutant produced significantly reduced levels of ST (less than 15% of wild type) when cultured in liquid GMM for 48 hours. This coincides with the relative gene expression values detected via qPCR analysis which show reductions in expression (60–90% reduction) of the transcriptional activator gene *aflR* and the two structural genes *stcQ* and *stcE* of the ST gene cluster in all mutants (Fig. 3c).

The relative expression values of genes belonging to the PN and TQ clusters were also tested. Expression of the PN genes *acvA*, *aatA* and *ipnA* was significantly reduced (55–85% reduction) in all mutants in comparison to wild type (Fig. 3d). Expression values of the TQ genes *tdiA* and *tdiB* showed the most dramatic reductions in all mutants (90–95% reduction) in comparison to wild type (Fig. 3e). Taken together, these data suggest that HamE and the pheromone module proteins all contribute in a similar manner to regulate the expression of the velvet complex genes, allowing for subsequent regulation of sexual development and production of various SMs.

### HamE influences expression and phosphorylation of the pheromone module components

**Figure 4:**
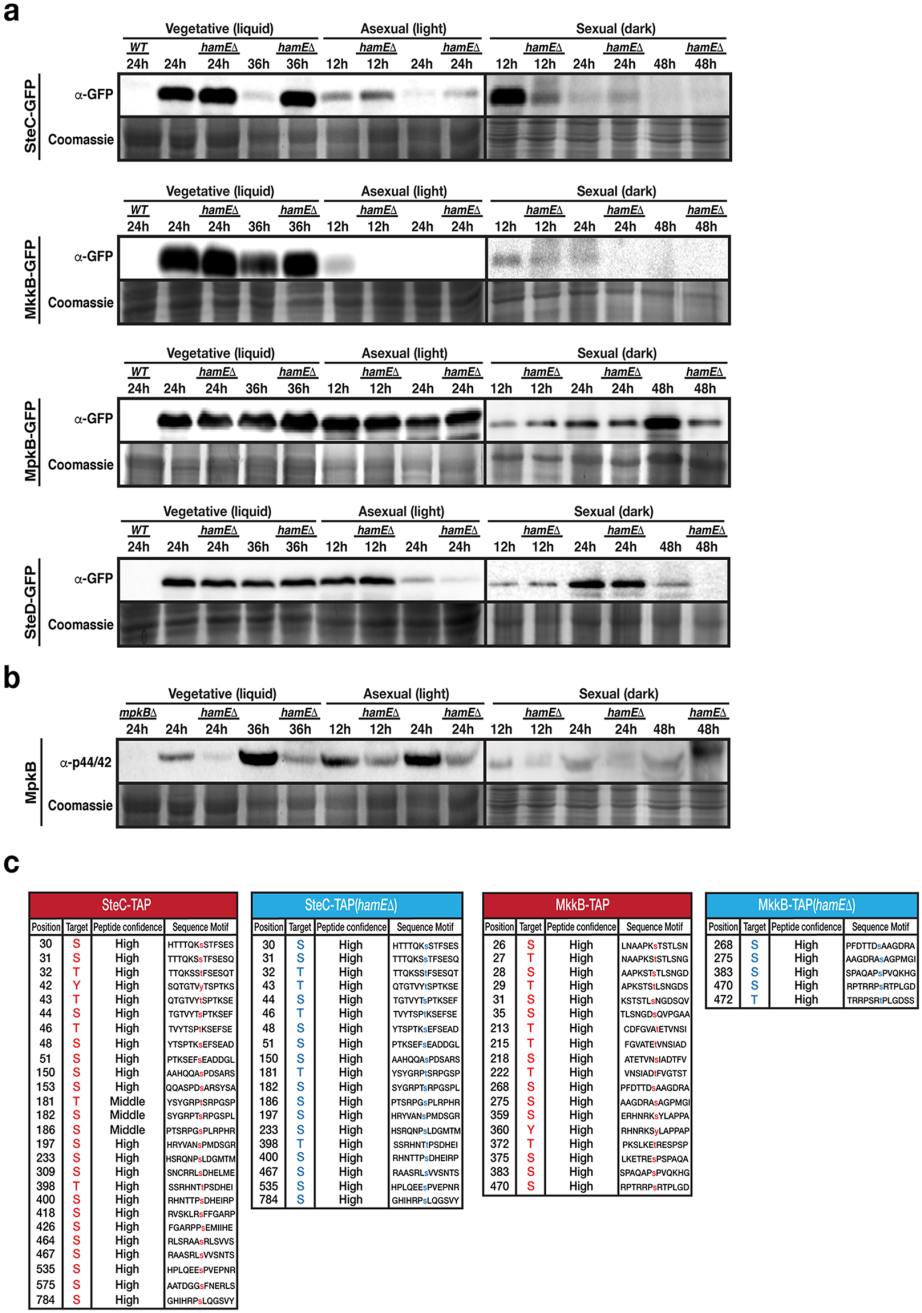
Expression levels and phosphorylation states of the pheromone module components in the presence and absence of *hamE*. (**a**) The expression levels of sGFP-tagged SteC, MkkB, MpkB and SteD fusion proteins were determined at various stages of development in the presence and absence of *hamE*. Vegetative cultures were grown in liquid GMM media. For asexual and sexual cultures, strains were initially grown for 24 hours vegetatively in liquid GMM media and then mycelia was transferred to GMM plates to be incubated in the light and dark respectively. 80 μg of each protein sample was loaded on 10% acrylamide gels and for loading controls, these gels were stained in 0.1% Coomassie Brilliant Blue R-250 dye and exposed using the G: BOX Chemi XRQ (Syngene). Sexual development samples (dark) were run in different gels separated from vegetative and asexual samples by a vertical black line. (**b**) Determination of the phosphorylation status of MpkB in the presence and absence of *hamE* using an anti-phospho-p44/42 antibody (Thr182/Tyr184). Sexual samples were run in different blots. SteC samples from part (**a**) were used for these blots. As a result, coomassie staining controls are the same as those for SteC samples. (**c**) Comparison of the phosphorylated residues of SteC and MkkB in the presence and absence of *hamE*. The tables represent the total phosphorylated residues and their amino acid positions detected by mass spectrometry using 4 independent TAP-tagged biological replicates of each strain. S (Serine), T (Threonine), Y (Tyrosine). Full-length blots in (**a**,**b**) are presented in Supplementary Fig. 3.

To investigate the influence of HamE on the expression of the pheromone module proteins, SteC, MkkB, MpkB and SteD were fused to GFP epitope tags and expressed in a strain lacking the *hamE* gene (Supplementary Fig S1). A time course immunoblotting was performed to compare the levels of expression of each protein in HamE(+) and HamE(−) backgrounds throughout different stages of development (Fig. 4a). This revealed dynamic expression profiles for each protein, with SteC, MkkB, MpkB and SteD all exhibiting differences to one another. This suggests that these proteins may play unique roles separate to those they perform as part of the pheromone module.

SteC was found to be upregulated at 24 hours of vegetative growth in both HamE(+) and HamE(−) backgrounds. However, at 36 hours of vegetative growth, it is evident that SteC is degraded in the wild type but expression is maintained in a HamE(−) background. Slight upregulation of SteC also occurs at 12 and 24 hours of asexual growth in the *hamE* mutant. Interestingly, at 12 hours of sexual induction, SteC is expressed in abundance in the wild type but expression is reduced in the HamE(−) background. MkkB was found to be strongly expressed during the early stages of vegetative growth (24 and 36 hours) with increased expression at 36 hours of growth in the *hamE* mutant. MkkB was also found to be readily degraded during the early stages of asexual and sexual development. MpkB was found to be expressed at all stages of development and deletion of *hamE* did not have any dramatic effects on MpkB expression. It can be noted that MpkB showed slightly reduced expression at 48 hours of sexual growth in the *hamE* mutant. SteD showed consistent expression during vegetative growth and early asexual development (12 hours), with the HamE(−) background exhibiting no effect on expression at these time points. Slight reductions can be observed in the *hamE* mutant at 24 hours of asexual growth and 48 hours of sexual growth.

Activation of the pheromone module is determined by it’s protein-protein interactions and phosphorylation states. Since HamE stably interacts with MkkB and MpkB, which are the two central kinases of the pheromone module, we decided to look at the phosphorylation states of MkkB and MpkB and also the MAP3K SteC. SteD was not used as it is the adaptor protein of the module. We first tested the phosphorylation of MpkB in HamE(+) and HamE(−) backgrounds during each stage of development (Fig. 4b). MpkB becomes dually phosphorylated at residues Threonine 182 and Tyrosine 184, allowing for it’s activation. By using an α-phospho-p44/42 MAPK antibody which detects phosphorylation at these residues, it was found that MpkB phosphorylation is significantly reduced in the *hamE* mutant at all stages of development. We then assessed whether HamE plays a role in the phosphorylation of SteC and MkkB. By performing TAP pulldowns of 4 biological replicates for TAP-tagged SteC and MkkB in HamE(+) and HamE(−) backgrounds, the total phosphorylation sites were combined in each strain for comparison (Fig. 4c, Supplementary Tables S5–10). A total of 26 phosphorylation sites were detected for wild type SteC and 19 sites were detected in the *hamE* mutant. 18 phosphorylation sites were detected for wild type MkkB but only 5 sites were detected in the *hamE* mutant. These data together underline complex modes of regulation for the pheromone module proteins and highlight potential roles of HamE in the regulation of kinase expression and phosphorylation, allowing for subsequent modulation of sexual development and secondary metabolism.

### Assembly and localisation of the pheromone module is not HamE-dependent

**Figure 5:**
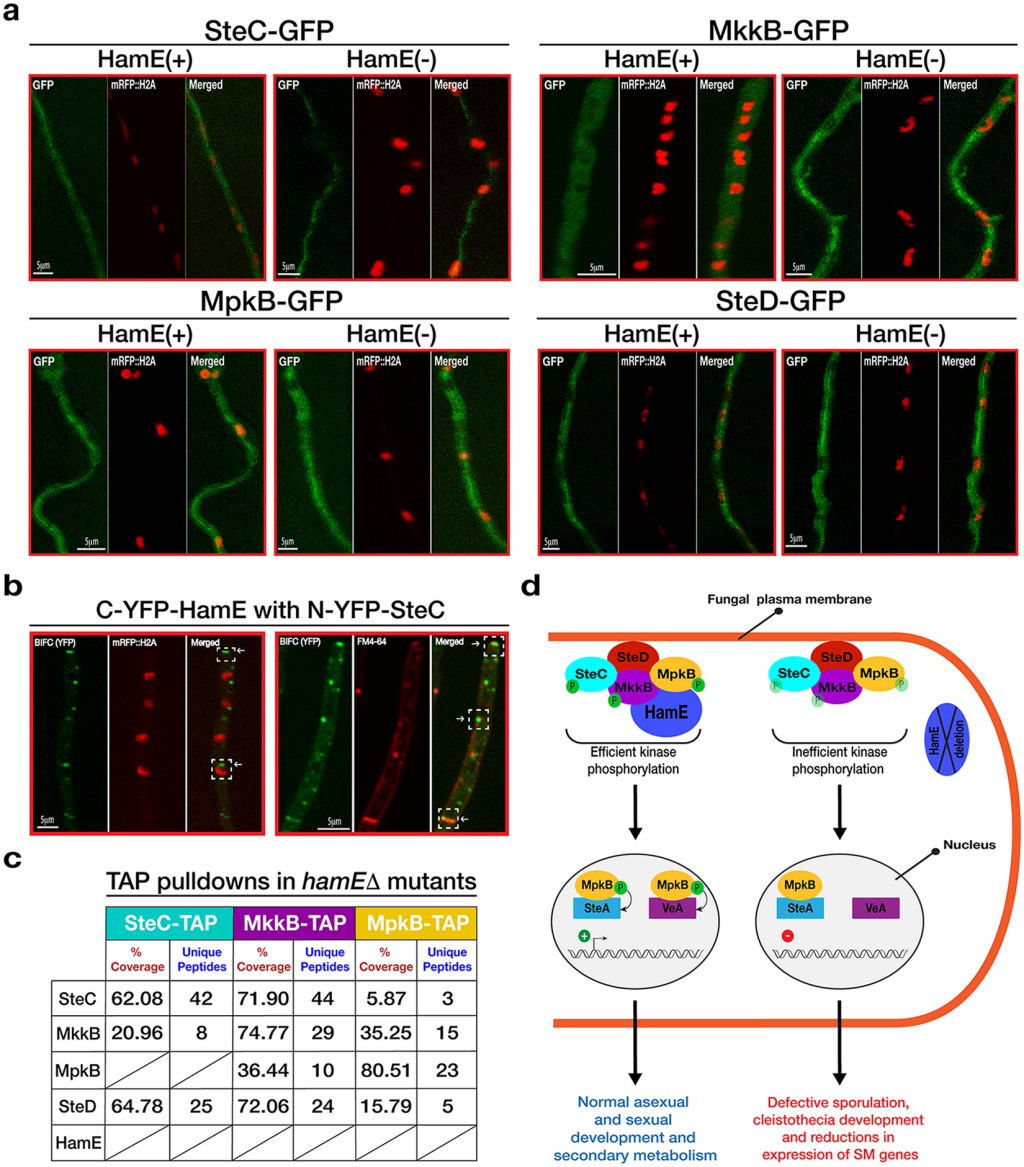
HamE co-localises with SteC but does not influence the sub-cellular localisations of kinases or complex assembly, (**a**) Sub-cellular localisations of the GFP-tagged pheromone module proteins at 16 hours of vegetative growth in the presence and absence of HamE. (**b**) BIFC showing interaction of C-YFP-HamE and n-YFP-SteC. White arrows indicate co-localisation of these two proteins at the hyphal tips, septa, plasma membrane and nuclear envelope. (**c**) TAP pulldowns of kinases in *hamE*Δ backgrounds at 24 hours of vegetative growth. (**d**) Schematic model of the pheromone module and the regulatory roles of HamE in kinase signalling, fungal development and secondary metabolism. HamE binds the kinases MkkB and MpkB and co-localises with the tetrameric complex of SteC-MkkB-MpkB-SteD at the plasma membrane, hyphal tips and nuclear envelope. HamE is required for efficient kinase phosphorylation, specifically MpkB. ‘P’ represents phosphate groups and inefficient kinase phosphorylation in the *hamE* mutant is represented by transparent phosphate groups. Efficient MpkB phosphorylation allows for MpkB to activate SteA and VeA to regulate both development and secondary metabolism, respectively.

To assess whether HamE influences sub-cellular localisations of the pheromone module proteins, GFP-fused kinases in HamE(+) and HamE(−) backgrounds were visualised via confocal microscopy (Fig. 5a). All members of the pheromone module, except for MpkB, showed cytoplasmic localization without nuclear enrichment. MpkB was nucleocytoplasmic as evident from colocalization of GFP and nuclear mRFP signals as published previously^36^. It is apparent from Fig. 5a that deletion of *hamE* does not influence localisation of these proteins in comparison to the HamE(+) strains. We then decided to test whether HamE is required for pheromone complex assembly by performing TAP pulldowns of kinases in *hamE*Δ backgrounds. Interestingly, it was shown that in a *hamE* mutant, the entire tetrameric complex is capable of assembling (Fig. 5c, Supplementary Tables S11–S13) and MpkB is capable of binding to transcription factor SteA (Supplementary Table S13). This signifies that MpkB is capable of translocating into the nucleus in a HamE-independent manner.

Considering that HamE was detectable at the hyphal tips, plasma membrane and nuclear envelope (Fig. 1e), BIFC was performed to test whether HamE co-localises with the kinase SteC. It was observed that HamE interacts with SteC at the hyphal tip, plasma membrane, septa and nuclear envelope (Fig. 5b). It has been previously shown^36^, via BIFC, that SteC also co-localises with MkkB, MpkB and SteD at the same sites, suggesting that HamE may also co-localise with the entire tetrameric complex at these sites.

## Discussion

MAP kinase modules are conserved signal transduction pathways in eukaryotes that regulate a range of processes. To regulate signalling of these MAP kinase modules, large multi-domain proteins known as scaffolds are utilised. Scaffolds often have binding domains for protein-protein interactions. As a result, scaffolds are capable of binding and promoting the interactions of at least two signalling proteins and have been shown to exhibit active regulatory roles^52^. The yeast Fus3 module and Ste5 scaffold represent a mechanistic paradigm for MAP kinase module signalling and regulation. However, in filamentous fungi, the lack of Ste5 homologs suggests a unique method of signal regulation in these pathways. Identification of the Ham5 scaffold in the *N*. *crassa* MAK-2 module^45,48^ has led to the hypothesis that Ham5 homologs may function as scaffolds in other filamentous fungi.

The *A*. *nidulans* pheromone module consists of the MAP3K SteC, MAP2K MkkB, MAPK MpkB and adaptor protein SteD^36^. In this study, we have identified the *A*. *nidulans* Ham5 homolog (HamE) and have provided evidence of scaffolding and regulatory roles for HamE in the pheromone module. HamE has been shown to physically interact with the kinases MkkB and MpkB (Fig. 1a, Supplementary Tables S2–S4) and is essential for efficient phosphorylation of each kinase, allowing for signal propagation to the nucleus. TAP pulldowns coupled to MS initially detected the uncharacterised HamE protein (AN2701) in purifications of SteC, MkkB and MpkB (Fig. 1a, Supplementary Tables S1–S3). Reciprocal BLAST searches of AN2701 confirmed that HamE is a homolog of *N*. *crassa* Ham5 and is a large protein with 6 WD40 repeats (Fig. 1c) which serve as a scaffolding domain allowing for protein-protein interactions^53^. TAP purifications of HamE resulted in detection of MkkB and MpkB (Fig. 1a, Supplementary Table S4). Although HamE was absent in purifications of SteC, HamE and SteC have been shown to co-localise at the hyphal tips, septa, cell membrane and nuclear envelope (Fig. 5b), suggesting that HamE and SteC may transiently interact and also that HamE may co-localise with the entire tetrameric complex at these sites.

This study has shown that the pheromone module proteins and HamE are crucial for the regulation of asexual sporulation, sexual cleistothecia formation and SM production in *Aspergillus nidulans* (Figs 2 and 3). In *A*. *nidulans*, the presence and absence of light induces asexual and sexual development respectively^29,54^. The velvet complex (VeA-VelB-LaeA) is required for the regulation of asexual and sexual development in response to light signals and is critical for the co-ordination of sexual development with secondary metabolism^32^. It has been shown that activation of MpkB in the pheromone module results in MpkB translocation into the nucleus where it activates SteA and VeA, promoting velvet complex assembly and coordination of sexual development and SM production^36^. The pheromone module mutants and HamE mutant exhibited 50–60% reduced sporulation (Fig. 2f) and were unable to produce cleisthothecia, forming only premature nests of Hulle cells (Fig. 2b,e). These data complement results observed for *ham5* mutants in *N*. *crassa*, which were shown to produce reduced levels of sexual reproductive structures known as protoperithecia^45^. In the *A*. *nidulans* mutants in this study, reduced expression of velvet complex genes (Fig. 3a), or presumably reduced phosphorylation of SteA may result in altered signalling dynamics in response to light signals, resulting in the reductions in SM production and sterile phenotypes observed. All mutants exhibited dramatic reductions in production of sterigmatocystin, penicillin and terrequinone A (Fig. 3), which are all, in turn, regulated by *laeA*^55,56^.

Phosphoproteomics analysis revealed that HamE contains at least 8 phosphorylation sites (Fig. 1c, Supplementary Table S5). The maximum protein coverage detected for HamE by MS was 46.5%, and so, it is likely that more phosphorylation sites exist. This data, coupled to the expression levels of HamE at different stages of development (Fig. 1d) suggest that HamE is a direct target of regulation. This could implicate HamE in higher order regulatory processes such as positive and negative feedback loops in the pheromone module as is the case for yeast Ste5^57,58^. In a *hamE* mutant, expression levels of the pheromone module proteins, particularly SteC, show dramatic changes (Fig. 4a). It was found that the intensity of MpkB phosphorylation is significantly reduced at all stages of development in the absence of *hamE* (Fig. 4b), which complements the reduced levels of MAK-2 phosphorylation observed in a *N*. *crassa ham5* mutant^45^. MS analysis also showed reductions in SteC and MkkB phosphorylation levels in the *hamE* mutant (Fig. 4c, Supplementary Tables S6–S9). These data suggest that HamE exhibits an active regulatory role and it is possible that HamE is required to catalytically unlock MpkB for phosphorylation by MkkB, which is evident for Ste5 in yeast^17^. This could explain why the tetrameric complex can assemble in a *hamE* mutant (Fig. 5c) but MpkB phosphorylation is inefficient and transcription factor activation does not occur.

In conclusion, this study has presented new insight on the organisation and signalling dynamics of the *A*. *nidulans* pheromone module. We propose the Ham5 homolog (HamE) as a regulatory scaffold for the pheromone module and provide evidence that HamE modulates the expression levels and phosphorylation states of each kinase, specifically MpkB. As a result, HamE is critical for signal transduction to the nucleus and regulation of both asexual and sexual development as well as production of various SMs (Fig. 5d). Understanding the molecular basis of signalling mechanisms in *A*. *nidulans* will allow for application of this knowledge to other filamentous fungi with biotechnological and medical relevance such as *Aspergillus flavus* and *Aspergillus fumigatus* since most filamentous fungal genomes encode orthologs of HamE, rather than Ste5. Characterisation of HamE homologs in these species may provide insight on the regulatory mechanisms involved in development as well as the production of clinically important SMs like aflatoxins and gliotoxin.

## Methods

### Strains, growth media and culturing conditions

Fungal strains used in this study are listed in Supplementary Table S14. The *Aspergillus nidulans* AGB551 (*veA*+) strain served as a wild type host for all deletions and epitope taggings. Various plasmids used for the knock-out and epitope tagging experiments are listed in Supplementary Table S15. Plasmids were cloned into Stellar (Clontech) and MACH-1 (Invitrogen) competent *Escherichia coli* cells and these cells were cultured in LB media (supplemented with 100 μg/ml ampicillin) and SOC media. For the growth of fungal strains, Glucose Minimal Media (GMM) was used. For asexual and sexual induction, fungal strains were cultured in liquid GMM for 24 hours and the mycelia was filtered through miracloth and transferred to GMM agar plates to be incubated in the light and dark respectively. For TAP experiments, fungal strains were cultured in complete medium.

### Phenotypic assays

Strains were point inoculated (5 × 10^3^ spores) in triplicate on GMM agar plates containing appropriate supplements. Plates were incubated in the presence of light for 4 days and the absence of light for 5 days to induce asexual and sexual development respectively. All incubations were performed at 37 °C. Stereomicroscopic images were captured using the Olympus szx16 microscope with Olympus sc30 camera. Digital pictures were taken and processed with the Cell Sens Standard software (Olympus). Quantifications of colony diameter, asexual conidiation and cleistothecia production were performed using three independent biological replicates. Bar charts represent the mean values ± s.d. *P*-values were calculated by performing unpaired Student’s *t*-tests (*P < 0.05; **P < 0.01; ***P < 0.001), using the Graphpad Prism Version 6.

### Tandem Affinity Purification (TAP), GFP-Trap and sample preparation for LC-MS protein identification

Isolation and preparation of TAP and GFP fusion proteins for mass spectrometry analysis was performed as explained in detail^32^. Detailed descriptions of methods used are given in supplementary information.

### Immunoblotting

For GFP-tagged proteins, mouse α-GFP antibody (SC-9996, SantaCruz) was used at 1:1,000 dilution in blocking solution (TBST with 5% milk). Secondary goat α-mouse (170–6516, Biorad) was used at 1:2,000 dilution in blocking solution. For the detection of SkpA, custom made rabbit α-SkpA was used at 1:1,000 dilution in blocking solution. For the detection of phosphorylated MpkB, rabbit α-phospho p44/42 (Cell Signalling Technology) was used at 1:1,000 dilution in TBST with 5% BSA. Goat α-rabbit (Biorad) was used as a secondary antibody for both SkpA and phosphorylated MpkB detection at 1:2,000 dilution in blocking solution.

### RNA extraction and quantitative real time PCR analysis

100 mg of mycelia was collected and mRNA was isolated according to the ‘RNeasy Plant Mini Kit’ protocol (Qiagen). mRNA was quantified according to the ‘Qubit RNA BR Assay Kit’ Protocol (Thermo Fisher). cDNA was synthesised from 1 μg of mRNA per strain using the ‘Transcriptor First Strand cDNA Synthesis Kit’ (Roche). qPCR reaction mixtures were prepared using LightCycler 480 SYBR Green I Master mix and a LightCycler 480 qPCR machine (Roche) was used to determine gene expression levels, using a Beta-tubulin (*benA*) control gene as a reference. Bar charts represent the mean data of two combined biological replicates and 6 combined technical replicates per strain, ± s.d.

### RP-HPLC analysis of Sterigmatocystin levels

Detailed information on culturing conditions and preparation of samples for RP-HPLC analysis is provided in supplementary information. 3 biological replicates were prepared per strain and data is presented as a bar chart, with the bars representing the mean ± s.d. *P*-values were calculated by performing unpaired Student’s *t*-tests (*P < 0.05), using the Graphpad Prism Version 6.

### Confocal microscopy

GFP and mRFP-tagged strains were inoculated (5 × 10^3^ spores) in 500 μl liquid GMM media with supplements and cultured in Lab-Tek Chambered Coverglass W/CVT (Thermo Scientific) for 16 hours at 30 °C. Localisations of the proteins were captured using the Zeiss LSM 510 META inverted confocal microscope.

## Electronic supplementary material

Supplementary Information
