# Supplementary material for: The HamE scaffold positively regulates MpkB phosphorylation to promote development and secondary metabolism in Aspergillus nidulans
Source: Sci Rep. 2018 Nov 8;8:16588. doi: 10.1038/s41598-018-34895-6 (PMC6224500; doi:10.1038/s41598-018-34895-6)
Supplement: Supplementary file 1 — Supplementary Information [file 41598_2018_34895_MOESM1_ESM.docx]

**Supplementary Information**

**The HamE scaffold positively regulates MpkB phosphorylation to promote development and secondary metabolism in *Aspergillus nidulans***

Dean Frawley^1^, Betim Karahoda^1^, Özlem Sarikaya Bayram^1^, Özgür Bayram^1,2,*^

^1^Biology Department, Maynooth University, Maynooth, Co. Kildare, Ireland

^2^Maynooth University Human Health Research Institute

^*^Correspondence: [ozgur.bayram@mu.ie](mailto:ozgur.bayram@mu.ie)

**Supplementary methods**

**Strains, growth media and culturing conditions**

The fungal strains used in this study are listed in Table S14. The *Aspergillus nidulans* AGB551 (*veA+*) strain served as a wild type host for all deletions and epitope taggings. Various plasmids used for the knock-out and epitope tagging experiments are listed in Table S15. Plasmids were cloned into Stellar (Clontech) and MACH-1 (Invitrogen) competent *Escherichia coli* cells and these cells were cultured in LB media (1% tryptone, 0.5% yeast extract, 1% NaCl), supplemented with 100μg/ml ampicillin and SOC media (2% tryptone, 0.5% yeast extract, 10mM NaCl, 2.5mM KCl, 10mM MgCl_2_, 10mM MgSO_4_, 20mM glucose). For the growth of fungal strains, Glucose Minimal Media (GMM) (6g/L NaNO_3_, 0.52g/L KCl, 1.52g/L KH_2_PO_4_, 10g/L Glucose, 0.24g/L MgS0_4_, 0.1% trace element solution) was used. This was supplemented with biotin (25μg/L), pyridoxine (10mg/L), uracil (1g/L), uridine (0.25g/L), pyrithiamine (0.1mg/L) (Sigma) and nourseothricin sulphate (100mg/L) (Gold Bio). For asexual and sexual induction, fungal strains were initially cultured in liquid GMM for 24 hours and then the mycelia was filtered through miracloth and transferred to solid GMM plates containing 2% agar to be incubated in the light and dark respectively. For TAP experiments, fungal strains were cultured in complete medium (GMM media ingredients with the addition of 1g/L tryptone, 2g/L peptone and 1g/L yeast extract).

**Plasmid Construction**

Details of all plasmids used in this study are given in Table S15 and all oligonucleotide sequences are provided in Table S16. The Lasergene Seqbuilder software was used to design all plasmid maps in silico. For all cloning experiments, pUC19 (Fermentas) digested with a *Sma*I restriction enzyme (Thermo Scientific) was used. For construction of deletion fragments, 1-1.5 Kb 5’ UTR and 3’ UTR flanking regions of gene of interest were PCR-amplified from genomic DNA with respective oligonucleotides. These UTR fragments were fused by fusion PCR to a selection marker (*ptrA, pyroA*) that was PCR-replicated from a plasmid containing the marker of interest. These three fragments were then cloned in the *Sma*I site of pUC19 by In-Fusion HD Cloning enzyme (Clontech) to create a circular plasmid.

To create the *sgfp* and *ctap* fusion constructs, 1.5-2Kb of the gene ORF (with stop codon removed) and 3’ UTR sequences were PCR-amplified from genomic DNA and fused to either *sgfp* or *ctap* epitope tags connected to selection markers (*natR, ptrA*). All epitope tags were fused to the gene ORFs at the C-terminal ends. These three fragments were then cloned into the *Sma*I site of pUC19.

**Transformation of bacterial and fungal cells**

150μl of competent Stellar/MACH-1 *E. coli* cells were added to ligated circular plasmid constructs on ice and left to incubate for 30 minutes. Samples were heat shocked at 42^o^C for 1 minute and immediately put back on ice. 800μl SOC media was added and samples were left to incubate on a shaker (180 RPM, 1 hour, 37^o^C). Samples were centrifuged for 1 minute (13,000 RPM), the supernatant was removed and bacterial cells were spread on LB agar plates containing ampicillin. Plates were left to incubate at 37^o^C for 16 hours.

For transformation of fungal cells, strains were cultured for 24 hours at 37^o^C in liquid GMM. Mycelia was filtered through sterile miracloth and washed three times with citrate buffer (150mM KCl, 580mM NaCl, 50mM sodium citrate, pH5.5). Mycelia was transferred to sterile flasks and incubated in 20ml citrate buffer containing 500mg glucanase (Novozymes) and 240mg lysozyme (Serva) for 100 minutes at 30^o^C (50-60 RPM). Protoplasts were filtered through sterile miracloth into a 50ml falcon tube and pre-chilled STC1700 (1.2M sorbitol, 10mM Tris pH5.5, 50mM CaCl_2_, 35mM NaCl) was used to make the volume up to 50ml. Samples were inverted multiple times and left on ice for 10 minutes. Samples were centrifuged at 2,600 RPM for 15 minutes at 4^o^C. Supernatant was removed and pellet was resuspended in 1ml STC1700 buffer. The volume was made up to 50ml with STC1700 buffer and samples were centrifuged using the same settings. Supernatant was removed and pellet was resuspended in 1ml STC1700. Protoplasts were separated into two 150μl aliquots, with 1μg of linear DNA or 10μg of circular plasmid DNA being added to one and no DNA being added to the other, to act as a negative control. Samples were incubated on ice for 30 minutes. PEG4000 (60% PEG4000, 10mM Tris pH7.5, 50mM CaCl_2_) was added to each sample three times (2 x 250μl and 1 x 850μl aliquots). Following each addition, samples were inverted 15-20 times. Samples were left to incubate in a rack on top of ice for 30 minutes. STC1700 was added to each falcon to make the volume up to 15ml and samples were inverted. Falcons were centrifuged at 2,600 RPM for 15 minutes at 4^o^C. Supernatant was discarded, the pellet was resuspended and protoplasts were inoculated on GMM agar plates containing 1.2M sorbitol.

**Hybridization techniques**

The Lasergene SeqBuilder software was used to design the 5’ and 3’ UTR probes for hybridisation and allowed for selection of appropriate restriction enzymes for digesting genomic DNA. Probes were synthesised and labelled with Digoxigenin-11-UTP (PCR DIG Probe Synthesis Kit: Roche), using either 5’ or 3’ UTRs as templates and the respective oligonucleotides. Fungal genomic DNA was isolated from mycelia using the ‘Zymo Research Fungal/Bacterial Miniprep Kit’. 700ng was digested overnight with a suitable restriction enzyme and was separated on a 0.7% agarose gel at 100 Volts for 90 minutes. The gel was washed 3 times on a shaker at room temperature in the following solutions (0.25M HCl for 10 minutes, 0.5M NaOH/1.5M NaCl for 25 minutes and 1.5M NaCl/0.5M Tris for 30 minutes). The DNA fragments were transferred and UV cross-linked (UV Stratalinker 1800) to a nylon membrane (Amersham Hybond^TM^-N^+^, GE Healthcare). The membrane was washed twice with 2x SSC (Saline Sodium Citrate) buffer and dried for 5 minutes at 70^o^C. The membrane was incubated in a rotating tube at 42^o^C in pre-hybridisation buffer for 1 hour on a rotator. The probe was then added and left to incubate overnight. The next day, the membrane was left on the rotator and was washed with 2x SSC/0.1% SDS solution for 5 minutes, followed by 2 washes with pre-heated 0.1x SSC/0.1% SDS for 20 minutes. The membrane was washed with 20ml DIG buffer 1, followed by incubation in 15ml DIG buffer 2 for 30 minutes. Alkaline phosphatase conjugated anti-DIG fab fragment (Roche 11093274910) was then added to the DIG buffer 2 (1:10,000 dilution) and left to incubate for 1 hour. 2 washes with 20ml wash buffer were performed for 15 minutes, followed by an incubation in 10ml DIG buffer 3 for 5 minutes. For chemiluminescent detection, CDP Star substrate (Roche) was added to the membrane and the membrane was exposed using the G:BOX Chemi XRQ (Syngene).

**Protein extraction and immunoprecipitation of fusion proteins**

Protein crude extracts were isolated from either vegetative cultures or mycelia that had been transferred to solid agar plates. Mycelia was broken using liquid nitrogen and protein extracts were prepared by re-suspending the broken mycelia in protein extraction buffer (300mM NaCl, 50mM Tris-HCl pH 7.5, 10% glycerol, 1mM EDTA, 0.1% NP-40) that had been supplemented with 1mM DTT, 1X cOmplete EDTA- free protease inhibitors (Roche), 1mM benzamidine, 0.5mM PMSF and 1X phosphatase inhibitors (1mM NaF, 0.5mM sodium orthovanadate, 8mM β-glycerol phosphate) immediately prior to use. Bradford assays were performed to determine protein concentrations and 80-100μg of protein extract was run on SDS gels with various percentages (10-15%).

For the immunoprecipitation of GFP fusion proteins, protein crude extracts were isolated from vegetative cultures grown for 24 hours in either liquid GMM or complete media. Per protein sample, 10μl GFP-Trap sepharose (Chromotek) was washed twice with 190μl protein extraction buffer. The GFP-Trap beads were then resuspended in 50μl protein buffer and added to 1ml protein extract. This mixture was left to incubate on a rotator for 3 hours at 4^o^C. Samples were placed in a magnetic rack and the supernatant was discarded. Beads were washed twice with 1ml protein extraction buffer (without supplements) and were then washed with the same buffer containing 1mM DTT. All liquid was removed and the beads were stored at -80^o^C until further use.

For TAP-tagged proteins, strains were cultured vegetatively for 24 hours in complete media and transferred to GMM agar plates for asexual and sexual induction. B250 buffer (250mM NaCl, 100mM Tris-HCl pH7.5, 10% glycerol, 1mM EDTA and 0.1% NP-40) was used to extract proteins from mycelia. The same supplements as described above were added to this buffer prior to use. Approximately 12-15ml of B250 buffer was used per 30ml of ground mycelial powder. Samples were centrifuged for 25 minutes at 16,000 RPM at 4^o^C. The protein extracts were transferred to new falcon tubes and 200μl of NHS-activated magnetic beads (Pierce) coupled to IgG from rabbit serum (Sigma) was added to each sample. Samples were left to incubate on a rotator at 4^o^C for 4 hours. Samples underwent three washes with the following buffers: WB250 (250mM NaCl, 40mM Tris-HCl pH8, 0.1% NP-40), WB150 (150mM NaCl, 40mM Tris-HCl pH8, 0.1% NP-40) and TCB (WB150 with 500mM EDTA). To WB250 and WB150 buffers, supplements were added prior to use as described for B250 buffer, while the only supplements added to TCB before use were 1mM DTT and 0.5mM PMSF. After washes, all liquid was removed and beads were resuspended in 1ml TCB buffer and transferred to E-cups. 20μl of TEV protease (AcTEV^TM^: Invitrogen) was added to the samples and left to incubate on a rotator at 4^o^C overnight. The next day, 50μl Magnezoom^TM^-CAM beads (Bioworld) was resuspended in 200μl CBB buffer (WB150 with 1mM MgOAc, 2mM CaCl_2_, 1mM imidazole, 10mM β-mercaptoethanol) per sample. In 15ml falcons, 6ml CBB buffer was added, followed by 200μl CAM bead solution, 8μl CaCl_2_ and 1ml of the TEV-treated protein extracts. These mixtures were then left to incubate on a rotator at 4^o^C for 2 hours. Beads were then placed on a magnetic rack and the supernatant was discarded. Beads were washed with CBB and transferred to E-cups. Beads were collected again, followed by another wash with CBB. All liquid was removed and beads were stored at -80^o^C until further use.

**Sample preparation for LC-MS protein identification**

Isolated GFP and TAP-tagged proteins were resuspended in 50mM ammonium bicarbonate. 1μl of 0.5M DTT was added and samples were incubated at 56^o^C for 20 minutes. 2.7μl of iodoacetamide (0.55M) was added and samples were incubated in the dark for 15 minutes. 1μl of 1% (w/v) ProteaseMAX (Promega) was added followed by addition of 1μl trypsin (1μg/μl) (Promega). Samples were left to incubate overnight at 37^o^C. The next day, 1μl of Trifluoroacetic acid (TFA) was added to each and samples were vortexed briefly and left to incubate for 5 minutes at room temperature. Beads were collected on a magnetic rack and the supernatant was transferred to new tubes. The supernatants were centrifuged at 13,000 RCF for 10 minutes and dried in a speedy-vac for 3 hours. Samples were stored at -20^o^C until further use.

Peptide samples were resuspended in 20μl resuspension buffer (0.5% TFA) and sonicated for 3 minutes, followed by a brief centrifugation. ZipTip C_18_ pipette tips (Millipore) were used to purify peptide samples prior to mass spectrometric analysis. To equilibrate the ZipTips, a wetting solution (0.1%, 80% acetonitrile) was aspirated 5 times, followed by aspiration of an equilibration buffer (0.1% TFA) 5 times. ZipTips were then used to pipette the peptide samples up and down 15-20 times. Then, the equilibration buffer was aspirated again 5 times, followed by elution of the peptides via aspiration of an elution buffer (0.1% TFA, 60% acetonitrile) 5 times into a new E-cup. This solution was dried in a speedy-vac for 2 hours and peptide samples were stored at -20^o^C.

Immediately prior to loading, peptide samples were resuspended in 10μl resuspension buffer (0.02% TFA) and 8μl was added to mass spectrometry vials (VWR). Samples were loaded on a high resolution quantitative LC-MS mass spectrometer (Thermo Fisher Q-Exactive). LC-MS identifications of peptides and their phosphorylation sites were performed using the Proteome Discoverer Daemon 1.4 software (Thermo Fisher). Unique peptides were determined by isolating only those that do not appear in any of the wild type samples.

**Immunoblotting**

For all immunoblots, protean membranes (0.45μm pore size, GE Healthcare) were incubated in blocking solution (5% (w/v) non-fat dry milk solution in 1X TBS with 0.1% Tween 20) for 1 hour at room temperature with gentle shaking. For the detection of GFP-tagged proteins, mouse α-GFP antibody (SC-9996, SantaCruz) was used at 1:1,000 dilution in blocking solution for 2 hours at room temperature. Secondary goat α-mouse (170-6516, Biorad) was used at 1:2,000 dilution in blocking solution for 1 hour at room temperature. For the detection of SkpA, custom made rabbit α−SkpA was used at 1:1,000 dilution in blocking solution for 2 hours at room temperature. For the detection of phosphorylated MpkB, rabbit α−phospho p44/42 (Cell Signalling Technology) was used at 1:1,000 dilution in 5% BSA solution for 2 hours at room temperature. Goat α-rabbit (Biorad) was used as a secondary antibody for both SkpA and phosphorylated MpkB detection at 1:2,000 dilution in blocking solution for 1 hour at room temperature. After each antibody incubation, membranes were washed three times with 1X TBST (0.1% Tween 20) for 5 minutes. For visualisation of all membranes, Luminata Crescendo Western HRP Substrate (Millipore) was added and membranes were exposed using the G:BOX Chemi XRQ (Syngene). For all membranes, 80-100μg of protein was loaded in each lane. For coomassie staining loading controls, proteins were run on 10% SDS gels and incubated in fixing solution (50% methanol, 10% glacial acetic acid) for 1 hour with gentle shaking. Gels were then incubated in staining solution (0.1% Coomassie Brilliant Blue R-250, 50% methanol, 10% glacial acetic acid) for 20 minutes, followed by incubation in de-staining solution (40% methanol, 10% glacial acetic acid) solution. This solution was renewed 3 times before exposure of gels using the G:BOX Chemi XRQ (Syngene).

**RNA extraction and quantitative real time PCR analysis**

Strains were inoculated in duplicate in 40ml of liquid GMM at a concentration of 5 million spores/ml and incubated for 48 hours on a shaker at 37^o^C. Mycelia was filtered through miracloth and washed with DEPC buffer (0.1% DEPC in 1X PBS) three times. 100mg of mycelia was collected in RNAse-free E-cups and mRNA was isolated according to the ‘RNeasy Plant Mini Kit’ protocol (Qiagen). mRNA was quantified according to the ‘Qubit RNA BR Assay Kit’ Protocol (Thermo Fisher). To synthesise cDNA, 1μg of mRNA was used for each strain and the ‘Transcriptor First Strand cDNA Synthesis Kit’ (Roche) was used. The final 20μl cDNA solutions were made up to 100μl with PCR-grade water and stored at -20^o^C until further use.

For qPCR, cDNA of each duplicate strain was inoculated in triplicate in 96-well plates (Life Science Products). Plates were loaded in a LightCycler 480 qPCR machine (Roche) and the cycle parameters were as follows: Pre-incubation (95^o^C, 10 minutes), Amplification [40 cycles] (95^o^C-10 seconds, 60^o^C-20 seconds, 72^o^C-10 seconds), Melting curve (65^o^C to 97^o^C with continuous fluorescence readings). Advanced relative quantification was used to determine the levels of gene expression in each strain, using a Beta-tubulin (*benA*) control gene as a reference for all strains. Relative expression analysis was performed by using the LightCycler 480 software. Bar charts represent the mean data of two combined biological replicates and 6 combined technical replicates per strain, ± s.d.

**RP-HPLC analysis of Sterigmatocystin levels**

Strains were inoculated in duplicate in 40ml of liquid GMM at a concentration of 5 million spores/ml and incubated for 48 hours on a shaker at 37^o^C. Mycelia was filtered through miracloth and 25ml of the liquid media was collected in a 50ml falcon tube. 25ml of chloroform was added to each and samples were briefly vortexed, followed by incubation on a rotator at 4^o^C for 1 hour. Samples were centrifuged at 4,000 RPM for 15 minutes at 4^o^C. 20ml of the lower phase of each sample was transferred to new 50ml falcon tubes and left to evaporate in a fume hood. Samples were resuspended in 2ml chloroform, transferred to 2ml E-cups and dried in a speedy-vac for 1 hour. Samples were resuspended in 200μl methanol and were loaded on a Shimadzu RP-HPLC with a photodiode array detector (PDA). 20μl of samples were injected onto a Luna**®**Omega 5μm Polar C18 (LC column 150 x 4.6m.m) and separated across a water:acetonitrile gradient with 0.1% (v/v) TFA. A sterigmatocystin standard (Sigma) was used at 12.5μg/ml concentration. Gradient conditions of 5-100% acetonitrile over 30 minutes with a flow rate of 1ml/minute were used with a PDA detection at 254nm. 3 biological replicates were prepared for each strain and the data is presented as a bar chart, with the bars representing the mean ± s.d. *P*-values were calculated by performing unpaired Student’s *t*-tests (**P*<0.05), using the Graphpad Prism Version 6


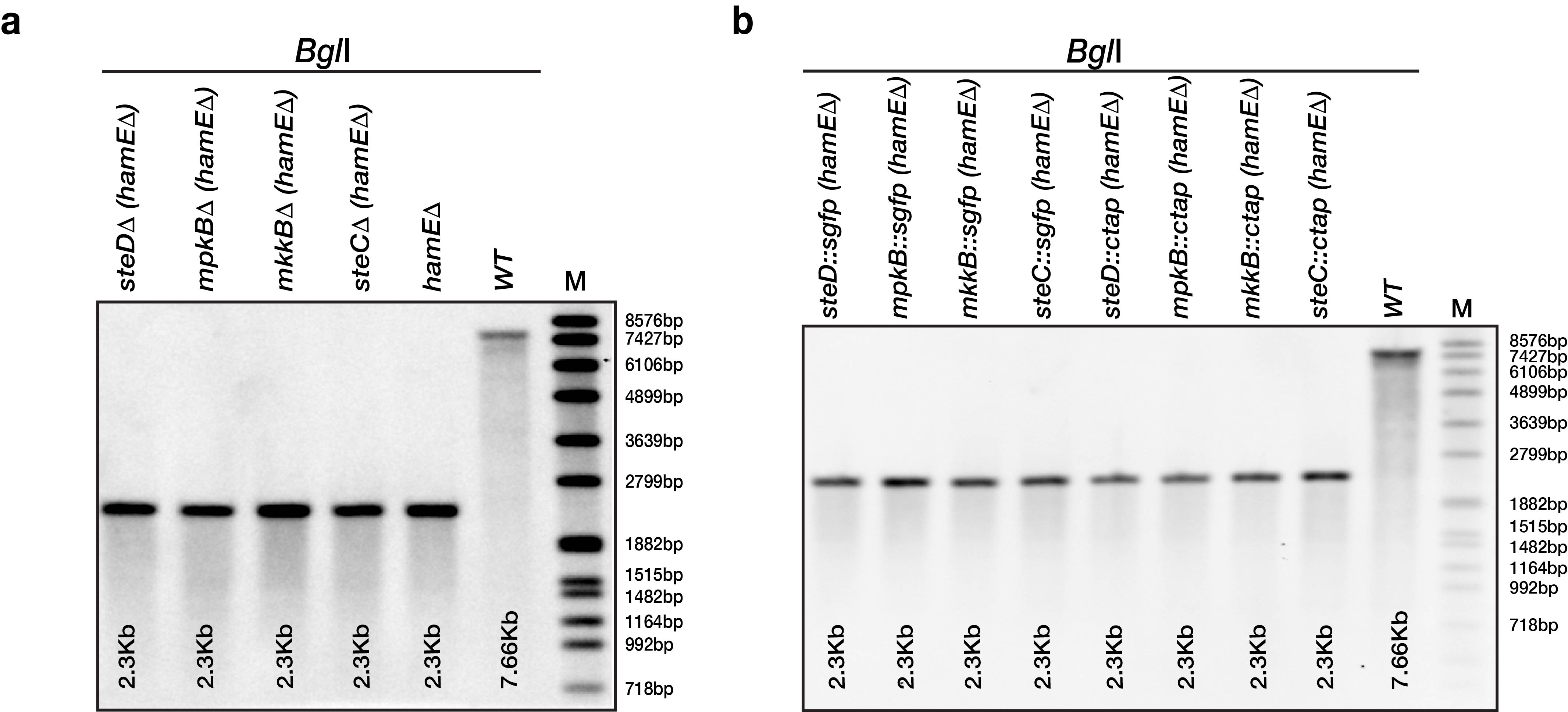


**Supplementary** **Figure 1. Confirmation of *hamE* deletions in TAP, GFP and kinase deletion strains**

(a) Southern hybridizations of *hamE*Δ in kinase deletion background strains. M: Molecular marker in basepairs (bp). Sizes of the bands are shown for the wild type strain and the deletion strains and are in accordance with theoretical maps. The *Bgl*I restriction enzyme was used to digest genomic DNA and a 5’ UTR DIG-labelled probe was used for detection. (b) Southern hybridizations of *hamE*Δ in *ctap* and *sgfp* fused kinase backgrounds.


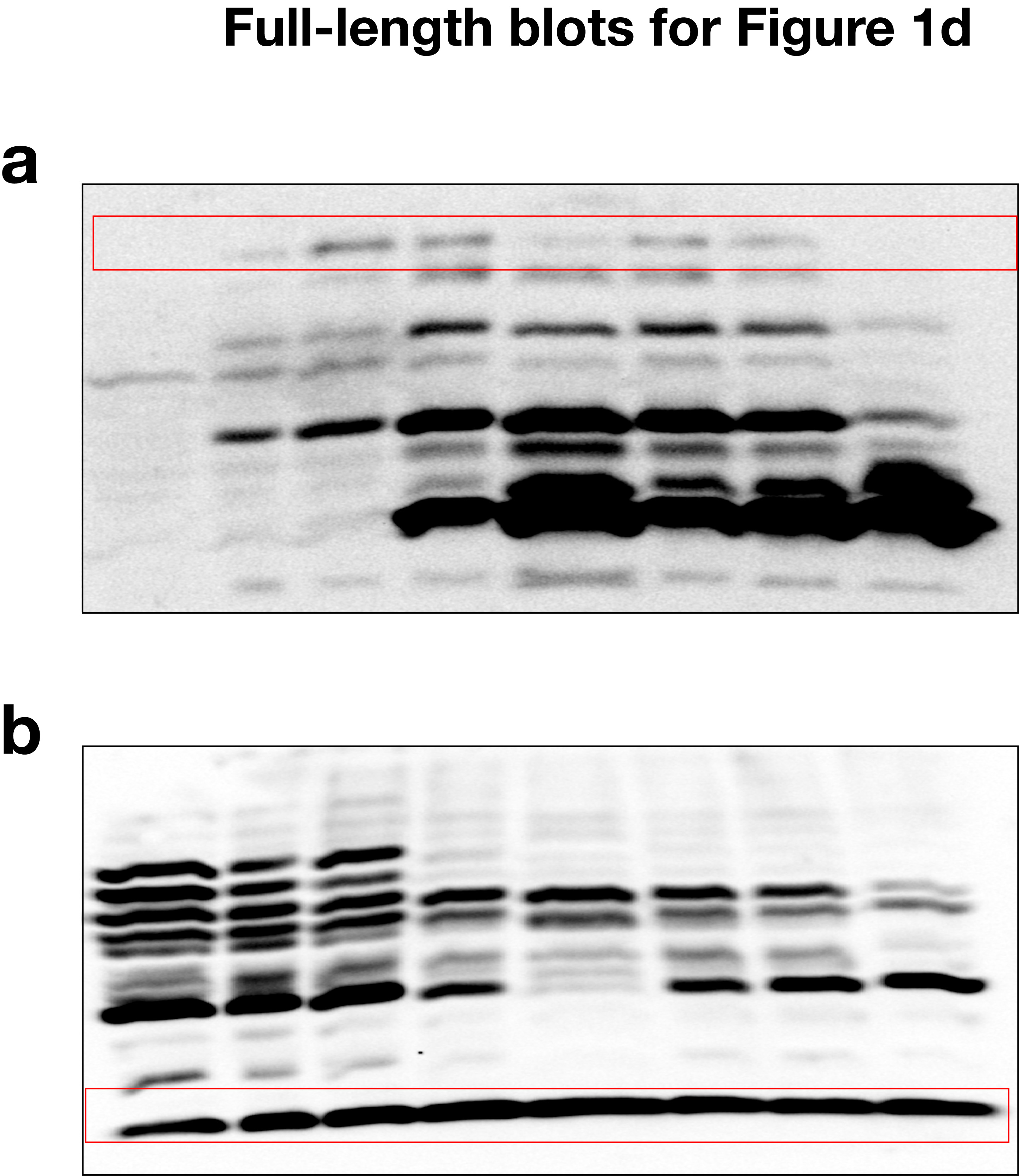


**Supplementary** **Figure 2. Full-length blots used to generate Figure 1d.**


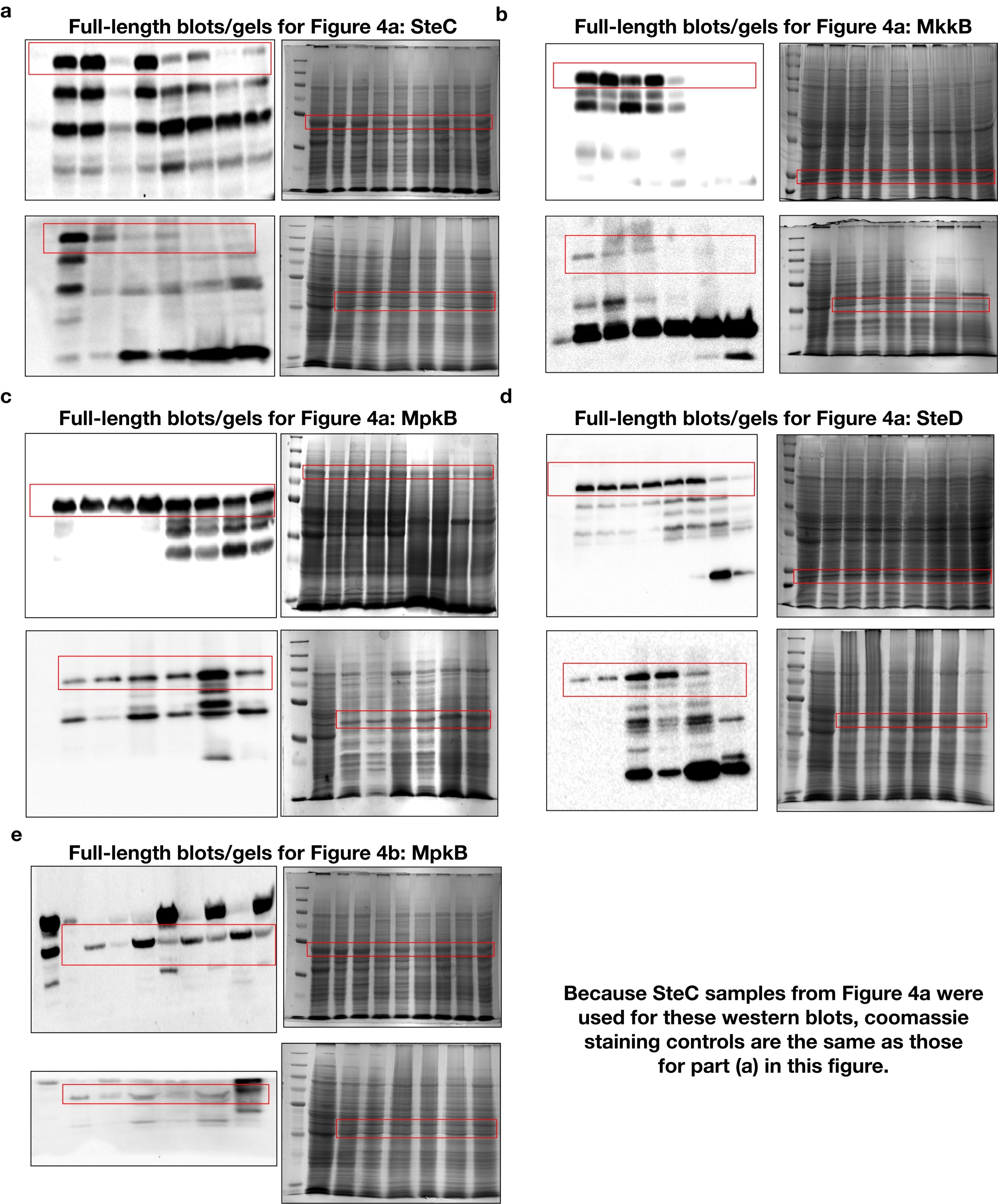


**Supplementary** **Figure 3. Full-length blots used to generate Figure 4a and b.**

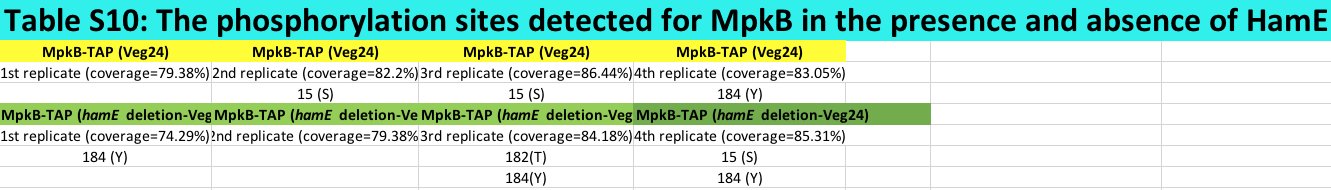

| **Supplementary Table S14.** Fungal strains created or used in this study | | |  |  |  |
| --- | --- | --- | --- | --- | --- |
| **Strain** | **Genotype** | **Plasmid used** | | **Reference** |  |
| AGB551 | *nkuAΔ::argB, pyrG89, pyroA4, veA+* | Not applied | | ^1^ |  |
| AGB586 | *mkkB∆::ptrA, nkuA∆::argB, pyrG89, pyroA4,* *veA1* | Fusion PCR | | ^1^ |  |
| AGB590 | *mkkB::sgfp::natR; nkuA****∆****::argB, pyroA4, pyrG89, veA1* | Fusion PCR | | ^1^ |  |
| AGB591 | *mrfp::h2A-pyrG; mkkB::sgfp::natR; nkuA∆::argB, pyroA4, pyrG89, veA1* | pME3858 in AGB590 | | ^1^ |  |
| AGB597 | *mkkB::ctap::natR; nkuA∆::argB, pyroA4, pyrG89, veA+* | Fusion PCR | | ^1^ |  |
| AGB650 | *steD∆::ptrA , nkuA∆::argB, pyrG89, pyroA4, veA+* | Fusion PCR | | ^1^ |  |
| AGB654 | *mpkB::sgfp::natR; nkuA****∆****::argB, pyrG89, pyroA4, veA+* | Fusion PCR | | ^1^ |  |
| AGB655 | *pgpdA::mrfp::h2A-pyrG; mpkB::sgfp::natR; nkuA∆::argB, pyrG89, pyroA4, veA+* | pME3858 in AGB654 | | ^1^ |  |
| AGB656 | *mpkB::ctap::natR; nkuA∆::argB, pyroA4, pyrG89, veA+* | Fusion PCR | | ^1^ |  |
| AGB657 | *steD::sgfp::natR; nkuA∆::argB, pyrG89, pyroA4, veA+* | Fusion PCR | | ^1^ |  |
| AGB659 | *steD::ctap::natR; nkuA∆::argB, pyroA4, pyrG89, veA+* | Fusion PCR | | ^1^ |  |
| SteC-GFP | *steC::sgfp::natR; nkuA∆::argB, pyroA4, pyrG89, veA+* | pOB478 in AGB551 | | This Study |  |
| SteC-TAP | *steC::ctap::natR; nkuA∆::argB, pyroA4, pyrG89, veA+* | pOB479 in AGB551 | | This Study |  |
| SteC-DEL | *steC∆::ptrA, nkuA∆::argB, pyrG89, pyroA4, veA+* | pOB483 in AGB551 | | This Study |  |
| MpkB-DEL | *mpkB∆::ptrA, nkuA∆::argB, pyrG89, pyroA4, veA+* | Fusion PCR | | This Study |  |
| HamE DEL-1 | *hamE∆::pyroA; nkuA∆::argB, pyrG89, pyroA4, veA+* | pOB451 in AGB551 | | This Study |  |
| HamE-GFP | *hamE::sgfp::natR; pyrG89, pyroA4, veA+* | pOB456 in HamE DEL-1 | | This Study |  |
| HamE-GFP-mRFP | *hamE::sgfp::natR; ^p^gpdA::mrfp::h2A::AfpyroA, pyrG89, pyroA4, veA+* | pOB340 in HamE-GFP | | This Study |  |
| HamE-TAP | | *hamE::ctap::natR; pyrG89, pyroA4, veA+* | pOB457 in HamE DEL-1 | | This Study |
| ANDF1 | | *steC::ctap::natR; hamE∆::pyroA; nkuA∆::argB, pyroA4, pyrG89, veA+* | pOB451 in SteC-TAP | | This Study |
| ANDF2 | | *mkkB::ctap::natR; hamE∆::pyroA; nkuA∆::argB, pyroA4, pyrG89, veA+* | pOB451 in AGB597 | | This Study |
| ANDF3 | | *mpkB::ctap::natR; hamE∆::pyroA; nkuA∆::argB, pyrG89, pyroA4, veA+* | pOB451 in AGB656 | | This Study |
| ANDF4 | | *steD::ctap::natR; hamE∆::pyroA; nkuA∆::argB, pyrG89, pyroA4, veA+* | pOB451 in AGB659 | | This Study |
| ANDF5 | | *steC::sgfp::natR; hamE∆::pyroA; nkuA∆::argB, pyroA4, pyrG89, veA+* | pOB451 in SteC-GFP | | This Study |
| ANDF6 | | *mrfp::h2A-pyrG; mkkB::sgfp::natR; hamE∆::pyroA, nkuA∆::argB, pyroA4, pyrG89, veA+* | pOB451 in AGB590 | | This Study |
| ANDF7 | | *pgpdA::mrfp::h2A-pyrG; mpkB::sgfp::natR; hamE∆::pyroA, nkuA∆::argB, pyrG89, pyroA4, veA+* | pOB451 in AGB654 | | This Study |
| ANDF8 | | *steD::sgfp::natR; hamE∆::pyroA, nkuA∆::argB, pyrG89, pyroA4, veA+* | pOB451 in AGB657 | | This Study |
| ANDF9 | | *steC∆::ptrA, hamE∆::pyroA, nkuA∆::argB, pyrG89, pyroA4, veA+* | pOB451 in SteC DEL | | This Study |
| ANDF10 | | *mkkB∆::ptrA, hamE∆::pyroA, nkuA∆::argB, pyrG89, pyroA4, veA+* | pOB451 in AGB586 | | This Study |
| ANDF11 | | *mpkB∆::ptrA, hamE∆::pyroA, nkuA∆::argB, pyrG89, pyroA4, veA+* | pOB451 in MpkB DEL | | This Study |
| ANDF12 | | *steD∆::ptrA, hamE∆::pyroA, nkuA∆::argB, pyrG89, pyroA4, veA+* | pOB451 in AGB650 | | This Study |
| ANDF13 | | *hamE∆::ptrA; pyrG89, pyroA4, nkuA∆::argB, veA+* | pOB341 in AGB551 | | This Study |
| ANDF14 | | *hamE∆::ptrA; mrfp::h2A-pyroA; pyrG89, pyroA4, nkuA∆::argB, veA+* | pOB207 in ANDF13 | | This Study |
| ANDF15 | | *steC::sgfp::natR; hamE∆::ptrA, nkuA∆::argB, pyroA4, pyrG89, veA+* | pOB341 in SteC-GFP | | This Study |
| ANDF16 | | *steD::sgfp::natR; hamE∆::ptrA, nkuA∆::argB, pyrG89, pyroA4, veA+* | pOB341 in AGB657 | | This Study |
| ANDF17 | | *steC::sgfp::natR; ^p^gpdA::mrfp::h2A::AfpyroA, pyroA4, pyrG89, veA+* | pOB340 in SteC-GFP | | This Study |
| ANDF18 | | *steD::sgfp::natR; ^p^gpdA::mrfp::h2A::AfpyroA, nkuA∆::argB, pyrG89, pyroA4, veA+* | pOB340 in SteD-GFP | | This Study |
| ANDF19 | | *steC::sgfp::natR; hamE∆::ptrA, ^p^gpdA::mrfp::h2A::AfpyroA, pyroA4, pyrG89, veA+* | pOB341 in ANDF15 | | This Study |
| ANDF20 | | *steD::sgfp::natR; hamE∆::ptrA, ^p^gpdA::mrfp::h2A::AfpyroA, nkuA∆::argB, pyrG89, pyroA4, veA+* | pOB341 in ANDF16 | | This Study |

| **Supplementary Table S15.** Plasmids created or used in this study | |  |
| --- | --- | --- |
| **Plasmid** | **Description** | **Reference** |
| pUC19 | *E. coli* cloning plasmid with *bla* (ampicillin resistance gene) gene | Thermo Fisher |
| pOB207 | *pgpdA::mrfp::h2A::pyroA* (histone 2A) in *Kpn*I site of pSK379 | This Study |
| pOB340 | *bioA5ORF::AfpyroA::pgpdA::mrfp::bioA3ORF* (histone 2A) with *bla* (*E.coli*) and *AfpyroA* (*A. nidulans*) | This Study |
| pOB341 | *hamE* (AN2701) deletion *with ptrA* in *Sma*I site of pUC19 | This Study |
| pOB451 | *hamE* (AN2701) deletion *with pyroA* in *Sma*I site of pUC19 | This Study |
| pOB456 | *hamE::sgfp::natR* cassette with *Pme*I site in *Sma*I site of pUC19  complementation | This Study |
| pOB457 | *hamE::ctap::natR* cassette with *Pme*I site in *Sma*I site of pUC19  complementation | This Study |
| pOB478 | *steC::sgfp::natR* cassette with *Pme*I site in *Sma*I site of pUC19 | This Study |
| pOB479 | *steC::ctap::natR* cassette with *Pme*I site in *Sma*I site of pUC19 | This Study |
| pOB483 | *steC* deletion with *ptrA*  in *Sma*I site of pUC19 | This Study |
| pME3858 | *pgpdA::mrfp::h2A* (histone 2A) with *pyrG* marker | ^1^ |

| **Supplementary Table S16.** Oligonucleotides created or used in this study | |  |
| --- | --- | --- |
| **Designation** | **Sequence in 5' > 3' direction** | **Size (basepairs)** |
| 3422-A (*mkkB* 5UTR forward) | CTC GGG CGC TCA TCG TGT GTT G | 22 |
| 3422-B (*mkkB* 5UTR nest) | CTT GCA ATG GGA CAA GCG ACG | 21 |
| 3422-C (*mkkB* 5UTR reverse with *ptrA* tail) | CTT TTA CAT TTC GTT ACC AAT GGG ATC CCG TAA TCA ATT GGC GAC GGC GAC TGA AGA TTG | 60 |
| 3422-D (*mkkB* 3UTR forward with *ptrA* tail) | CAA GAA AGA CAG TAT AAT ACA AAC AAA GAT GCA AGA CCT CTA AAC TAT TCA TGG GCC CC | 59 |
| 3422-E (*mkkB* 3UTR nest) | CCA CTA GCC GAT GAA CGA GTA TTC | 24 |
| 3422-F (*mkkB* 3UTR reverse) | GAG CCT CTG TTG TAG TGG GTA GAG | 24 |
| OZG314 (*mkkB* 3UTR forward with tail for natR) | GCA GGC GCT CTA CAT GAG CAT GCC CTG CCC CTG ACC TCT AAA CTA TTC ATG GGC CCC | 57 |
| OZG380 (*mkkB* 5UTR reverse with GFP tail) | GCC CTT GCT CAC CAT ACC ACC GCT ACC ACC GAG GGC CCC CAT ATG GTC GCC GC | 54 |
| OZG382 (*mkkB* 5UTR reverse with TAP tail) | CTT TTT CCA TCT TCT CTT ACC ACC GCT ACC ACC GAG GGC CCC CAT ATG GTC GCC GC | 56 |
| OZG443 (*mpkB* 5UTR forward) | CCC AGA AGT CCC AGG CCA GTT C | 22 |
| OZG444 (*mpkB* 5UTR nest) | CAA GAG ATC ATT CTT GAG GCA AAA G | 25 |
| OZG445 (*mpkB* 5UTR reverse with *ptrA* tail) | CAT TTC GTT ACC AAT GGG ATC CCG TAA TCA ATT CTG CTG CAC CAT GTT GAC TGG | 54 |
| OZG446 (*mpkB* 3UTR forward with *ptrA* tail) | GAC AGT ATA ATA CAA ACA AAG ATG CAA GAT GTC ATC ACA GTT CTG ATT TAC GAG | 54 |
| OZG447 (*mpkB* 3UTR nest) | GCT GAC GGC AAT ATA GAA TCA TAC | 24 |
| OZG448 (*mpkB* 3UTR reverse) | CGA GGC GTT TGG GGA GAC GCT GAG | 24 |
| OZG470 (*steD* 5UTR forward) | GAC CAT CCA GAG GCG GTA ACG | 21 |
| OZG471 (*steD* 5UTR nest) | GTC GAA GAA TTT GCA TAT CGA TTA TC | 26 |
| OZG472 (*steD* 5UTR reverse with tail for *ptrA*) | CAT TTC GTT ACC AAT GGG ATC CCG TAA TCA ATT GAC GAG AGC GAG CTG ACG AC | 53 |
| OZG473 (*steD* 3UTR forward with tail for *ptrA*) | GAC AGT ATA ATA CAA ACA AAG ATG CAA GAA ACC ATC GCA GGG GCA TAT GC | 50 |
| OZG474 (*steD* 3UTR nest) | CGC GTG ATC TTT CAC GTA ACC G | 22 |
| OZG475 (*steD* 3UTR reverse) | CTC CGT AGG TGG AAT CCA AAC AG | 23 |
| OZG560 (*mpkB* 5UTR reverse with tail for GFP) | GCC CTT GCT CAC CAT ACC ACC GCT ACC ACC CCG CAT GAT CTC CTC GTA AAT CAG | 54 |
| OZG561 (*mpkB* 5UTR reverse with tail for TAP) | CTT TTT CCA TCT TCT CTT ACC ACC GCT ACC ACC CCG CAT GAT CTC CTC GTA AAT CAG | 57 |
| OZG562 (*mpkB* 3UTR forward with tail for natR) | GCG CTC TAC ATG AGC ATG CCC TGC CCC TGA AGA ATC AAG TGT CGA ATC TTG GAG TTG | 57 |
| OZG564 (*steD* 5UTR reverse with tail for GFP) | GCC CTT GCT CAC CAT ACC ACC GCT ACC ACC TAA AAC TCC GCC GGG AAG GTT G | 52 |
| OZG565 (*steD* 5UTR reverse with tail for TAP) | CTT TTT CCA TCT TCT CTT ACC ACC GCT ACC ACC TAA AAC TCC GCC GGG AAG GTT G | 55 |
| OZG566 (*steD* 3UTR forward with tail for natR) | GCG CTC TAC ATG AGC ATG CCC TGC CCC TGA AGG CAT GCG ACT TGG ATG AAG C | 52 |
| OZG928 (*hamE* 5UTR forward with tail for pUC19) | TTC GAG CTC GGT ACC CGT TTA AAC CGC AGC TGG TGG ACT TGG AAC | 45 |
| OZG929 (*hamE* 5UTR reverse with tail for ptrA) | GAT CCC GTA ATC AAT TAA TTC CGC CCG AAT CCG TGA C | 37 |
| OZG931 (*hamE* 3UTR reverse with tail for pUC19) | ACT CTA GAG GAT CCC CGT TTA AAC ACT GAC ACA TCT GCA GCG CAA G | 46 |
| OZG948 (*hamE* 5UTR reverse with tail for pyroA) | CAG CAT CTG ATG TCC AAT TCC GCC CGA ATC CGT GAC | 36 |
| OZG949 (*hamE* 3UTR forward with tail for pyroA) | GCC TCC TCT CAG ACA GGC TGA CTG CCT TTT GCT ACT CAC | 39 |
| OZG983 (*hamE* 5UTR reverse with tail for TAP/GFP) | ACC ACC GCT ACC ACC TAT ACG ACC ATC AGC ATC AGG AG | 38 |
| OZG984 (*hamE* 3UTR forward with tail for natR) | ATG CCC TGC CCC TGA CTT CCG TTT TAA TCT TTT TTC TTC TTT GCT G | 46 |
| OZG1019 (*steC* 5UTR with tail for pUC19) | TTC GAG CTC GGT ACC CGT TTA AAC CTG GGA ATC GGA GCG TGT TG | 44 |
| OZG1020 (*steC* 5UTR reverse with tail for GFP & TAP linker) | TAC CAC CGC TAC CAC CGG TAA GTG TTG TAG CAA GGA AG | 38 |
| OZG1021 (*steC* 3UTR forward with tail for natR) | CAT GCC CTG CCC CTG AAA TCC TTT ACG ATG TCG GAT AGA C | 40 |
| OZG1022 (*steC* 3UTR with tail for pUC19) | ACT CTA GAG GAT CCC CGT TTA AAC CAG CGT TTA ATT CAA CTT GAG CAT G | 49 |
| OZG1023 (*steC* 5UTR reverse with tail for ptrA) | GGA TCC CGT AAT CAA TTG GAT TAG TAG ATG GGC GTA TAG | 39 |
| OZG1024 (*steC* 3UTR forward with tail for ptrA) | CAA ACA AAG ATG CAA GAT GAA ATC CTT TAC GAT GTC GGA TAG | 42 |
| OSBRT1 (*laeA* 5UTR) | CAC AAC CAC TAC AGC TAC CAC | 21 |
| OSBRT2 (*laeA* 3UTR) | GCA ACC GCG TAT CTG GTC G | 19 |
| OSBRT7 (*ipnA* 5UTR) | GAG AGT AGC CCA GCA AAT CG | 20 |
| OSBRT8 (*ipnA* 3UTR) | GGC ACG AAT CGC AAG GTC C | 19 |
| OSBRT9 (*acvA* 5UTR) | GAC AAG GAC AAC CGT GAT G | 19 |
| OSBRT10 (*acvA* 3UTR) | GCA CAC CAT TAC TGC TAG AGG | 21 |
| OSBRT11 (*aatA* 5UTR) | CCA TTG ACT TCG CAA CTG GC | 20 |
| OSBRT12 (*aatA* 3UTR) | CGT ACG AGT GTT GAG CAT GAC | 21 |
| OSBRT13 (*tdiA* 5UTR) | CGA TGC CTG GAG TGC GAA TG | 20 |
| OSBRT14 (*tdiA* 3UTR) | GCC GTT GCT GTC AAT GAA CG | 20 |
| OSBRT15 (*tdiB* 5UTR) | GCT ACC TGC ACA CGA GCA GC | 20 |
| OSBRT16 (*tdiB* 3UTR) | GCG CTC TCA AAG TTC CGC TC | 20 |
| OSBRT57 (*aflR* 5UTR) | CCT TCG CTT CTT GAG GGT ATG G | 22 |
| OSBRT58 (*aflR* 3UTR) | GCA GTA GGA GTG GCT TGT GGT G | 22 |
| OSBRT68 (*stcE* 5UTR) | GCA TCT CGA TGT AGT GAT CG | 20 |
| OSBRT69 (*stcE* 3UTR) | CTA GTC GCC TGG AAC AGT AG | 20 |
| OSBRT70 (*stcQ* 5UTR) | GGT TGT AGC GTC TTT GCA ACG | 21 |
| OSBRT71 (*stcQ* 3UTR) | GAA CAT CGT TGC AGA ACG TGG | 21 |
| OSBRT76 (*veA* 5UTR) | CGA TCC AGA GCC TCT CAG AG | 20 |
| OSBRT77 (*veA* 3UTR) | GGT CAT CAT GAC CGA ACG AC | 20 |
| OSBRT78 (*velB* 5UTR) | CCT CCC ACA ATC GGA TAT TGC | 21 |
| OSBRT79 (*velB* 3UTR) | GGG ATC TTG ATT CCT TGG TTC | 21 |
| BK280 (*benA* 5UTR) | GAT GGC TGC CTC TGA CTT C | 19 |
| BK281 (*benA* 3UTR) | GCA TCT GGT CCT CAA CCT C | 19 |

**Supplementary References**

1 Bayram, O. *et al.* The Aspergillus nidulans MAPK module AnSte11-Ste50-Ste7-Fus3 controls development and secondary metabolism. *PLoS Genet* **8**, e1002816, doi:10.1371/journal.pgen.1002816 (2012).
